# Disrupting *Helicobacter pylori* Iron Homeostasis With Bismuth Nanodrug‐Mediated Nutritional Trap for Targeted Gastric Infection Therapy

**DOI:** 10.1002/advs.76621

**Published:** 2026-07-20

**Authors:** Tianye Fang, Jinzhe Tong, Feng Feng, Cong Liu, Chang Shu, Jiaying Zhu, Shibo Zhang, Shuyue Deng, Wanchao Zuo, Yuhan Song, Jun Yang, Yanmin Ju, Yingying Xing, Jianjun Dai

**Affiliations:** ^1^ School of Pharmacy China Pharmaceutical University Nanjing China; ^2^ School of Life Science and Technology China Pharmaceutical University Nanjing China; ^3^ Key Laboratory of Drug Quality Control and Pharmacovigilance China Pharmaceutical University Nanjing China; ^4^ Nanjing Institute for Food and Drug Control Nanjing China; ^5^ MOE Joint International Research Laboratory of Animal Health and Food Safety Key Laboratory of Animal Bacteriology Ministry of Agriculture College of Veterinary Medicine Nanjing Agricultural University Nanjing China

**Keywords:** gut microbiota, *H. pylori*, iron homeostasis, metal‐polyphenol network, nanodrug

## Abstract

*Helicobacter pylori* (*H. pylori*) is a leading cause of gastric cancer, but current antibiotic‐based therapies face challenges like low eradication rates, high recurrence, and intestinal flora disruption. Iron is critical for *H. pylori*’s survival and pathogenicity, as the bacterium relies on maintaining iron homeostasis for normal proliferation, metabolism, and colonization of the stomach. Inspired by this, we propose a “nutritional trap” strategy, using bismuth as “fake iron” to trick the *H. pylori*’s iron‐uptake system, triggering iron starvation. We then developed a bismuth‐based nanodrug (Bi‐TP@FU, TBF) modified with tea polyphenols and fucoidan for enhanced *H. pylori* targeting and gastric mucosal penetration. TBF targets and adheres to the surface of *H. pylori* by binding to the BabA protein, then induces iron starvation and metabolic disorders through competition between bismuth and iron, ultimately killing multidrug‐resistant strains without inducing resistance. Interestingly, TBF selectively eliminated pathogens while preserving probiotics, thus maintaining intestinal flora balance, which is superior to quadruple therapy. This novel oral TBF, which relies on a nutritional trap‐based bactericidal strategy, offers a new option for antibiotic alternative therapy of bacterial infections.

## Graphical abstract

A “nutritional trap” strategy centered on disrupting bacterial iron homeostasis is proposed. The synthesized Bi‐TP@FU (TBF) can target *Helicobacter pylori*, interfere with the bacterial iron uptake and utilization processes to disrupt its iron homeostasis, induce bacterial death, and meanwhile preserve the balance of the intestinal flora. This finding further offers a novel approach for antibiotic‐alternative therapies against *Helicobacter pylori* infection.

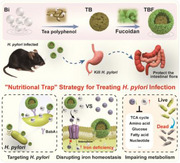

## 1 Introduction

*Helicobacter pylori* (*H. pylori*) is one of the bacteria with the highest infection rates globally and the most definitive risk factor for gastric cancer identified to date [1]. Consequently, clinical practice advocates for prompt treatment of patients upon confirmation of infection. However, with the rapid development of bacterial drug resistance, the eradication rate of current first‐line therapies based on antibiotics has significantly declined, even falling below 70% in some cases, while the recurrence rate remains as high as 10% [2, 3]. Meanwhile, the administration of antibiotics tends to disrupt the intestinal flora, posing a potential threat to patients’ health [4]. Against this backdrop, there is an urgent clinical need to explore safer and more effective alternative therapies to replace antibiotics.

During the process of infecting the human body, *H. pylori* must continuously acquire iron from the surrounding environment, which serves as the core survival resource determining whether *H. pylori* can successfully colonize the gastric mucosa, complete proliferation, and secrete virulence factors [5, 6, 7]. Existing research has indicated that intracellular iron deficiency in *H. pylori* disrupts its iron homeostasis, thereby disturbing core physiological processes, impairing pathogenicity, and ultimately leading to death [8, 9]. Notably, this iron homeostasis‐disrupting bactericidal mechanism fundamentally differs from antibiotics’ bactericidal mode of action and has not yet been reported for *H. pylori* treatment. Given this, a key question emerges: can we design a strategy that achieves efficient bactericidal effects by interfering with *H. pylori*’s iron homeostasis?

As a key component of the quadruple therapy for *H. pylori* eradication, bismuth ions (Bi^3+^) have been widely used in the treatment of gastric diseases. Notably, Bi^3+^ and iron ions (Fe^3+^) are both trivalent cations and tend to bind to key proteins in *H. pylori* [10]. These similarities may cause *H. pylori*’s iron‐uptake system to misrecognize Bi^3+^ as Fe^3+^ and actively take it up. More importantly, *H. pylori* lacks the relevant pathways for Bi^3+^ metabolism, which results in the accumulation of misabsorbed Bi^3+^ in the bacterium, leading to high‐concentration accumulation [11]. Unlike Fe^3+^, Bi^3+^ is a redox‐inert substance that cannot replace Fe^3+^ in regulating critical physiological processes within the bacterium [12]. This “similar‐yet‐dissimilar” property endows Bi^3+^ with the potential in treating *H. pylori* infection through iron deprivation.

Based on this, we propose a nutritional trap strategy by introducing Bi^3+^ as the fake iron to confuse *H. pylori*’s iron‐uptake system, inducing its death via iron starvation. However, the use of bismuth alone lacks targeting ability and also has difficulty reaching the bacterial colonization sites under the gastric mucosa [13]. These limitations weaken the bactericidal efficacy of Bi^3+^ in practice, explaining why bismuth agents in quadruple therapy still require combination with antibiotics for ideal effects [10, 13]. Certain natural organic macromolecules possess abundant active groups that bind metal ions into stable complexes, some even facilitating submucosal penetration [14, 15], targeting bacterial antigens [16], and maintaining intestinal flora homeostasis [17]. Therefore, bismuth‐based nanodrugs modified with natural organic macromolecules are expected to overcome the drawbacks of single‐use Bi^3+^ and more efficiently achieve precise treatment of gastric *H. pylori* infection.

**FIGURE 1:**
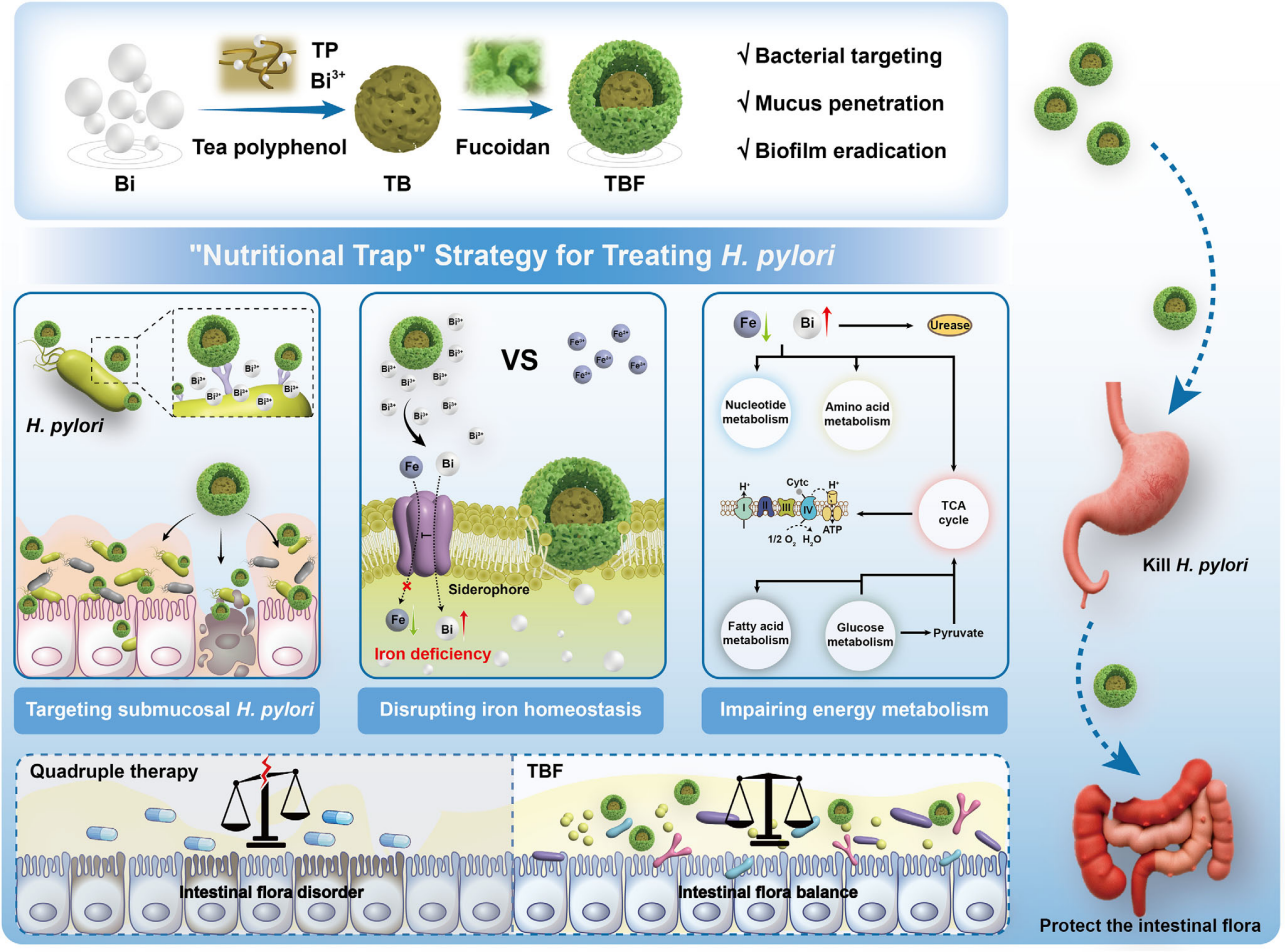
Illustration of the nutritional trap strategy for treating *H. pylori* infection. Bismuth was selected as a “false iron”, based on which the nutritional trap strategy was proposed. This strategy achieves efficient bactericidal effects by disrupting bacterial iron homeostasis while preserving intestinal flora balance.

In this study, we applied the novel strategy of disrupting *H. pylori* iron homeostasis via bismuth‐iron competition for treating *H. pylori* infection, successfully developing a bismuth‐based nanodrug (Bi‐TP@FU, TBF) by incorporating tea polyphenols (TP) and fucoidan (FU) (Figure 1). The experimental results confirmed that TBF exhibits significant anti‐*H. pylori* activity against both planktonic bacteria and biofilms, and shows superior therapeutic efficacy compared to the quadruple therapy in animal models of drug‐resistant bacterial infection. Our findings reveal that TBF can effectively penetrate the gastric mucosa barrier, specifically adhere to the bacterial surface through binding to the BabA protein, and ultimately induce bacterial death by competing with Fe^3+^ to disrupt iron homeostasis and interfere with vital metabolic processes. Notably, it displays potent antibacterial efficacy against multiple clinically isolated multidrug‐resistant strains, rarely induces drug resistance, and selectively eliminates pathogens while preserving murine probiotics, thus addressing a key limitation of conventional antibiotics. As a novel oral bismuth‐based nanodrug, TBF employs a nutritional trap strategy to target *H. pylori* iron homeostasis, presenting a promising antibiotic alternative for *H. pylori* therapy.

## 2 Results and Discussion

### 2.1 Antibacterial Activity Based on Nutritional Trap Strategy

**FIGURE 2:**
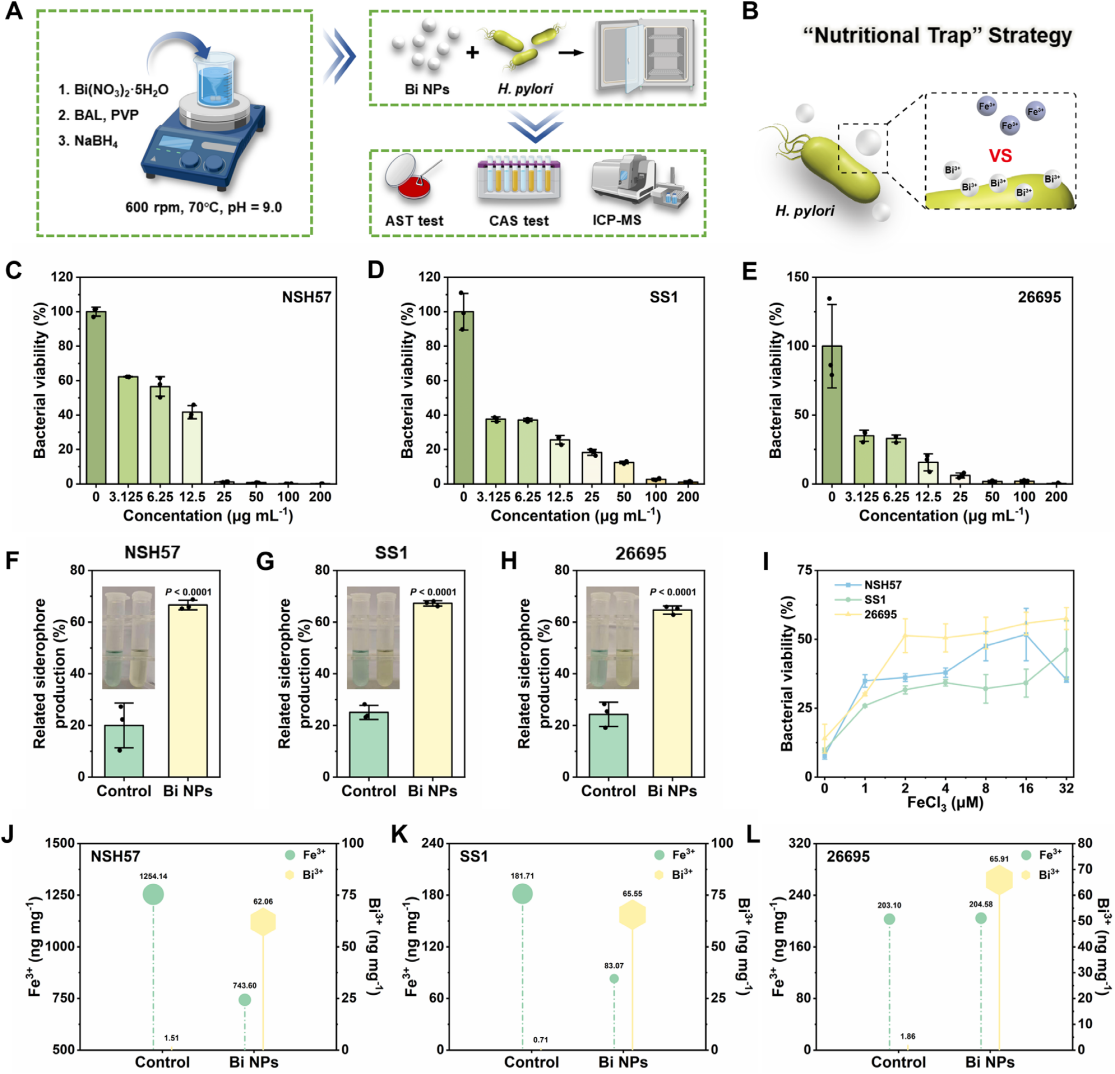
The antibacterial activity of Bi NPs. (A) Illustration of the Bi NPs preparation process. (B) Schematic diagram of the nutritional trap strategy. Bactericidal effect of different Bi NPs concentrations on *H. pylori* (C) NSH57, (D) SS1, and (E) 26695. (*n* = 3 biological replicates) The siderophore production of *H. pylori* (F) NSH57, (G) SS1, and (H) 26695. (*n* = 3 biological replicates, two‐sided Student's *t*‐tests) (I) Bacterial viability after Bi NPs treatment with the addition of FeCl_3_. (*n* = 3 biological replicates) Content of Fe^3+^ and Bi^3+^ in *H. pylori* (J) NSH57, (K) SS1, and (L) 26695 after Bi NPs treatment.

Bi NPs were synthesized via a convenient and gentle chemical reduction method to act as a novel nanodrug against *H. pylori* infection (Figure 2A) [18]. Based on this, we propose the nutritional trap strategy, which uses Bi^3+^ as “false iron” to confuse *H. pylori* and disrupt its iron homeostasis (Figure 2B). As shown in Figure 2C–E, Bi NPs exhibited certain antibacterial activity against *H. pylori* NSH57, SS1, and 26695, with a minimum inhibitory concentration (MIC) ranging from 25 to 100 µg mL^−1^.

To verify the association between this antibacterial activity and iron homeostasis, the chrome azurol S (CAS) assay was initially employed to determine the intracellular siderophore content in drug‐treated bacteria [19]. As shown in Figure 2F–H, all these *H. pylori* strains exhibited increased siderophore production to varying degrees after drug treatment. This result indicates that Bi NPs treatment can induce intracellular iron deficiency in bacteria, prompting them to increase siderophore secretion to compensate for this deficit. Subsequently, the effect of Bi NPs on bacterial iron metabolism was further verified by supplementing exogenous Fe^3+^ and comparing changes in bactericidal efficacy. After adding FeCl_3_, the bactericidal activity of Bi NPs against *H. pylori* was weakened (Figure 2I). Furthermore, inductively coupled plasma mass spectrometry (ICP‐MS) was used to detect intracellular ion contents. As shown in Figure 2J–L, the Bi^3+^ content in all three bacterial strains increased significantly after drug administration. Meanwhile, despite increased siderophore expression, the Fe^3+^ content in *H. pylori* NSH57 and SS1 still showed a marked decrease. These results confirm the successful implementation of the nutritional trap strategy.

### 2.2 Synthesis and Characterization of TBF

**FIGURE 3:**
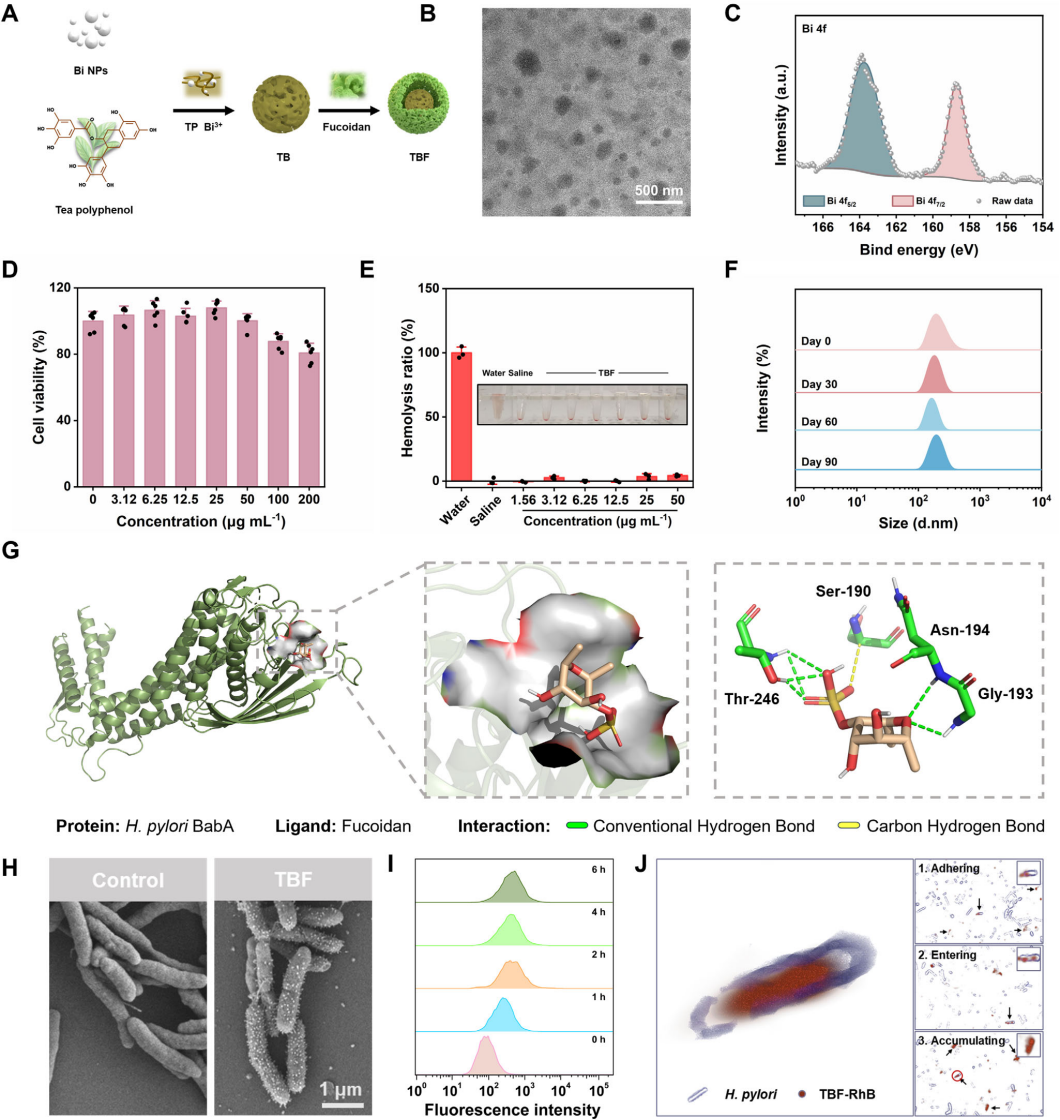
Characterization and anti‐*H. pylori* potential of TBF. (A) Illustration of the TBF preparation process. (B) Typical TEM images of TBF. (C) XPS spectrum of Bi 4f scan. (D) Cell survival of GES‐1 at different TBF concentrations. (*n* = 6 biological replicates) (E) Erythrocyte hemolysis at different TBF concentrations. (*n* = 3 biological replicates) (F) Particle size of TBF over a 3‐month period. (G) The predicted docking patterns between *H. pylori* adhesin protein BabA and TBF. (H) Representative SEM images taken after a 3 h co‐incubation of TBF with *H. pylori*. (I) Intra‐bacterial fluorescence intensity after co‐culture with fluorescently labeled TBF measured using multicolor a flow cytometry. (J) 3D rendered images of *H. pylori* merged with TBF fluorescence.

Two natural organic macromolecules, tea polyphenols and fucoidan, were employed to modify Bi NPs for further enhancing their targeting ability and safety. As shown in Figure 3A, a novel bismuth‐based nanodrug Bi‐TP@FU (TBF) was obtained via a one‐pot method [20]. Transmission electron microscopy (TEM) analysis showed that TBF was a spherical structure with uniform size and good dispersion (Figure 3B). The average size of TBF was measured to be around 135 nm in dynamic light scattering (DLS) (Figure S1). Such a uniform and homogeneous morphology, as well as a small size, can contribute to the sterilizing effect of TBF [21].

Ultraviolet–visible (UV–vis) was used to further observe the interactions between the components. Bi NPs had no characteristic absorption peak between 250 and 500 nm, but after tea polyphenols were coated on it, a new peak appeared at 271 nm, indicating that the charge transition between Bi^3+^ and tea polyphenols had taken place and a Bi^3+^ phenolic hydroxyl chelate had formed (Figure S2A) [22]. Meanwhile, the Fourier Transform infrared spectroscopy (FTIR) showed transmission peaks at 1616 cm^−1^ ascribed to the C─C/C═C stretching vibration. And the peaks at 1147 cm^−1^ as well as 1097 cm^−1^ can be attributed to the C─O stretching vibration. These characteristic peaks prove the successful binding of tea polyphenols. Besides, compared to the other two groups, TBF exhibited a glycan characteristic peak at 2928 cm^−1^ for C─H stretching vibration, indicating the successful encapsulation of the fucoidan (Figure S2B).

The valence state of Bi elements in TBF was analyzed by X‐ray photoelectron spectroscopy (XPS) (Figure 3C). The two peaks at 163.9 eV (Bi 4f_5/2_) and 158.8 eV (Bi 4f_7/2_) could correspond to Bi^3+^ [23]. In addition, X‐ray diffraction (XRD) was then employed to confirm the composition of TBF. As shown in Figure S3, the crystal face was same as Bi (JCPDS 85–1329) with some shifts. At the same time, a bulging peak appeared at 20°–40°, which was mainly due to the doping of tea polyphenols and fucoidan changing the crystal surface distance and affecting the crystal shape of TBF. These data further demonstrating the successful synthesis of TBF. Then, the biocompatibility of TBF was tested. As analyzed by the CCK‐8 assay kits, within the concentration range of 0–200 µg mL^−1^, the cellular activity of gastric mucosal epithelial cells (GES‐1) was not significantly affected by TBF, and the survival rate remained above 80% (Figure 3D). Additionally, no hemolysis has been observed after co‐incubating with TBF (Figure 3E), indicating excellent blood compatibility. In addition, the stability of the nanodrugs is equally crucial [24]. As shown in Figure 3F, the particle size of TBF did not change significantly during the monitoring process of up to 3 months, which proved that it could be stably preserved in the aqueous system for a substantial period. These results demonstrated that TBF exhibited favorable biocompatibility and biomedical application potential.

### 2.3 Interaction Between *H. pylori* and TBF

*H. pylori* secretes the adhesin BabA, which binds to the Lewis b antigen on the cell surface and enhances bacterial colonization [25]. Notably, this adhesion‐dependent colonization mechanism provides a specific target for TBF's targeted bacterial adhesion and capture capacity [26, 27]. Molecular docking results revealed that fucoidan formed a carbon‐hydrogen bond with GLU332 and conventional hydrogen bonds with GLN333, THR334, THR335, and HIS331 of BabA, exhibiting a binding energy of −5.8 kcal mol^−1^ (Figure 3G). This property endows TBF with the ability to target and capture *H. pylori*. During the experiment, significant sedimentation of bacteria was first observed following co‑incubation with TBF (Figure S4). Consistent with this observation, SEM images further confirmed that TBF adhered uniformly to the surface of *H. pylori* (Figure 3H). Fucoidan was subsequently selected as a potential blocking agent to mask the BabA protein [26]. The results revealed that free fucoidan alone exhibited negligible direct antibacterial activity against *H. pylori* (Figure S5A), whereas pre‑blocking BabA with fucoidan for 3 h markedly attenuated the subsequent bactericidal effect of TBF (Figure S5B). Combined with SEM observations and molecular docking results, these competitive inhibition assays indicate that TBF exerts its enhanced bactericidal activity via interaction with *H. pylori*, and this interaction is critical for mediating its antibacterial effects.

The ability of TBF to enter the bacteria was subsequently verified by flow cytometry, and the fluorescence values within the bacteria were saturated when TBF was incubated with the bacteria for 2 h (Figure 3I). To make this process clearer, optical diffraction tomography (ODT) was performed using the HT‐X1 equipment. ODT enables imaging of *H. pylori* by measuring the three‐dimensional (3D) refractive index (RI) of microscopic objects in a label‐free or fluorescence‐dependent manner in a wide area of cultured live bacteria [28]. The entry of TBF into the bacterial interior was observable in the 2D image (Figure S6), while 3D reconstruction results showed the sequential process of TBF: adhering to the bacterial surface, penetrating into the interior, and finally accumulating therein (Figure 3J). Together, these results demonstrate that TBF can target *H. pylori*, thereby exhibiting potential for efficient bacterial killing.

### 2.4 Antibacterial Activity of TBF

**FIGURE 4:**
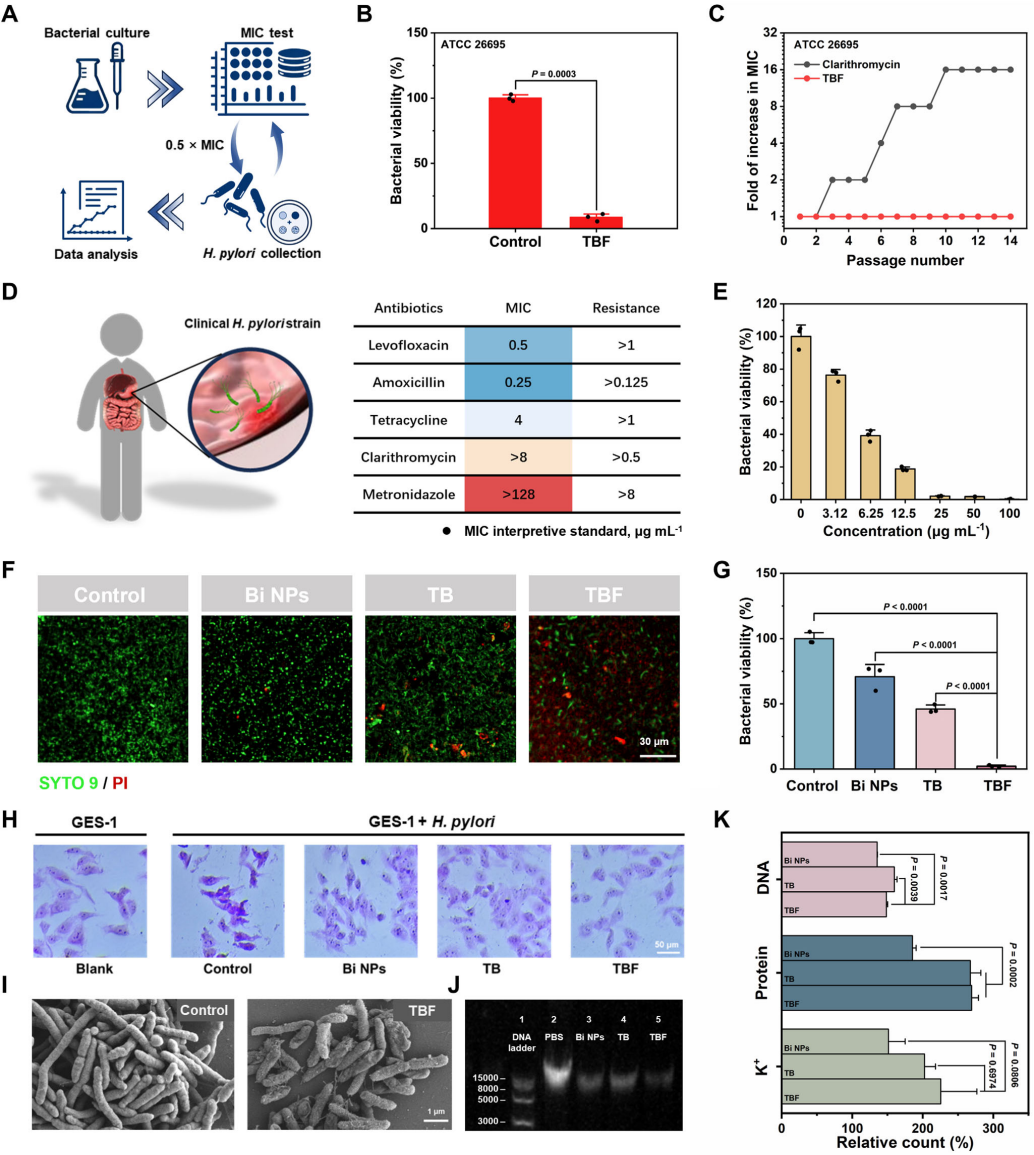
In vitro anti‐*H. pylori* activity of TBF. (A) Schematic of the cyclic antibacterial experiment process. (B) Bactericidal effect of TBF on *H. pylori* 26695. (*n* = 3 biological replicates, two‐sided Student's *t*‐tests) (C) Resistance properties of *H. pylori* 26695 to clarithromycin and TBF. (D) MIC values of the clinical isolate strain NSH57 against different antibiotics. (E) Statistics on the antimicrobial effects detected by the plate counting method with TBF. (*n* = 3 biological replicates) (F) Representative Live/Dead staining images of *H. pylori* after different treatments. (G) Statistics on the antibacterial effects of agar plates. (*n* = 3 biological replicates, one‐way ANOVA followed by post‐hoc Tukey's test) (H) Representative optical images of *H. pylori* adhering to the surface of GES‐1 cells after different treatments. (I) Representative SEM images taken after a 12 h co‐incubation of TBF with *H. pylori*. (J) Agarose gel electrophoresis images of *H. pylori* genomic DNA after different treatments. (K) Statistics on leakage of *H. pylori* K^+^, DNA, and proteins after different treatments. (*n* = 3 biological replicates, one‐way ANOVA followed by post‐hoc Tukey's test).

Traditional drugs such as antibiotics are prone to inducing bacterial resistance during long‐term or repeated use, which not only reduces therapeutic efficacy but also poses a serious threat to clinical anti‐infective treatment [29]. Therefore, we first verified the bactericidal properties of TBF on standard strains and judged whether TBF could induce drug resistance in *H. pylori* through cyclic antibacterial experiments (Figure 4A). As shown in Figure 4B, TBF produced a significant killing effect on 26695. And compared to the clinically used antibiotic clarithromycin, the bacteria remained sensitive to TBF even after 14 treatment passages, while the MIC of clarithromycin had risen to 16 times its initial value (Figure 4C).

Given that clarithromycin‐resistant *H. pylori* has been classified as a high‐priority pathogen among the 12 drug‐resistant bacteria identified by the World Health Organization as posing the greatest threat to health [30], we subsequently tested the MIC of several clinically isolated *H. pylori* strains with clinically recommended antibiotics and TBF. The experimental results showed that TBF had a good bactericidal effect on these strains even though they were all multidrug‐resistant bacteria (Table S1). On this basis, NSH57, the most resistant strain among these clinical isolates, was selected for subsequent experiments (Figure 4D). As shown in Figure 4E, a gradual enhancement in antimicrobial activity was observed with the increase in TBF concentration. And then, the effects of the different components were compared, and it was found that the addition of polyphenols and fucoidan significantly increased the antimicrobial effect of TBF (Figure 4F,G). On the one hand, it was due to the existence of a certain antimicrobial capacity of polyphenols themselves. On the other hand, fucoidan could bind to bacterial surface adhesin proteins, thus enabling TBF to target adhesion to the bacterial surface and obtain an improved bactericidal effect [31].

Furthermore, as TBF interacts with the adhesins of *H. pylori*, bacterial adhesion to GES‐1 cells was greatly reduced. Significant cell death occurred after only a few hours of co‐culture of *H. pylori* with GES‐1, whereas a reduction in bacterial adherence was clearly observed in the cells that underwent drug pretreatment, and the cells still retained their normal morphology (Figure 4H). Thus, it was demonstrated that the TBF could reduce *H. pylori* colonization to a certain extent. In addition, disruption of the bacterial membrane (Figure 4I), damage to genomic DNA (Figure 4J), and leakage of cellular contents (Figure 4K) were also observed, along with a decrease in bacterial metabolic activity (Figure S7) and mobility (Figure S8). The results of all these experiments demonstrated that *H. pylori* becomes less virulent and progressively dies after co‐incubation with TBF.

Subsequently, the role of iron‑homeostasis disruption in this process was validated via iron‑rescue assays and positive‑drug control experiments [32]. Supplementation with exogenous iron ions gradually mitigated the downstream cytotoxicity of TBF toward *H. pylori* (Figure S9). Meanwhile, deferasirox also induced similar toxic effects in *H. pylori* as TBF (Figure S10). These observations further solidify the critical role of iron deficiency in triggering downstream toxic effects in *H. pylori*.

### 2.5 Anti‐Biofilm Effect of TBF

**FIGURE 5:**
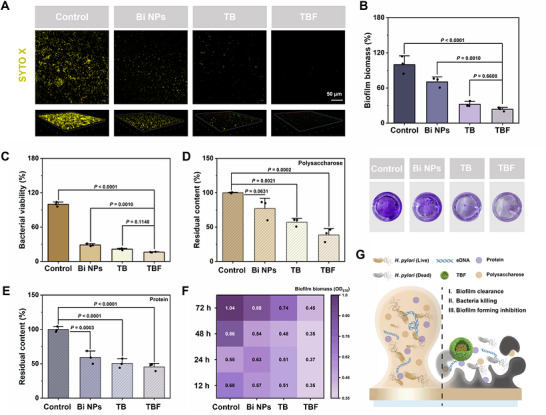
Biofilm removal and reformation inhibition of TBF. (A) Representative images of biofilm eDNA staining using SYTO X after different treatments. (B) Bacterial biomass of biofilms treated with different nanodrugs (Top) and representative images of crystalline violet staining (Bottom). (*n* = 3 biological replicates, one‐way ANOVA followed by post‐hoc Tukey's test) (C) The number of planktonic bacteria remaining in biofilms after different treatments. (*n* = 3 biological replicates, one‐way ANOVA followed by post‐hoc Tukey's test) Statistics of (D) polysaccharide and (E) protein content in EPS. (*n* = 3 biological replicates, one‐way ANOVA followed by post‐hoc Tukey's test) (F) The amount of biofilm biomass measured using crystal violet staining at 12, 24, 48 and 72 h after treatment with different nanodrugs. (G) Schematic diagram before and after biofilm eradication using TBF.

Biofilm formation by bacteria can greatly weaken the efficacy of antimicrobial drugs, and how to effectively remove biofilm has become a major difficulty in the actual clinical treatment process. Here, we tested the effect of TBF on the removal of biofilm at different stages. First, we used SYTO X to label extracellular DNA (eDNA) in extracellular polymeric substances (EPS) of biofilms, thus verifying the disruption and removal of immature biofilms by TBF. As shown in Figure 5A, the integrity of the biofilm was disrupted, and its thickness was thinned after treatment with different nanodrugs. Then, the biofilm was stained using crystal violet dye, and a significant reduction in the amount of biofilm biomass was observed after different treatments (Figure 5B). It is noteworthy that the TBF group showed better results than the other two groups.

At the same time, residual biofilm fragments were applied to the plate, and it was found that the number of remaining bacteria was also significantly reduced (Figure 5C). In addition, proteins and polysaccharides are two other major components of EPS. Therefore, the polysaccharide and protein contents in the biofilm after treatment were examined using the BCA method and the phenol‐sulfate method, respectively. It was found that the contents of both were significantly decreased (Figure 5D,E). The biofilm‑removing effect was further confirmed.

If the bacterial clearance is incomplete, it will still gather and regenerate biofilm through the secretion of adhesins and quorum sensing, which will lead to recurrent infections and make the actual treatment less effective [20, 33]. We therefore examined the entire process and found that biofilm regeneration occurred in all groups except the TBF group, which maintained the biofilm biomass at a low level within 72 h after treatment (Figure 5F). This may be due to the fact that fucoidan can bind to adhesins secreted by bacteria and interfere with bacterial self‐aggregation, thus preventing biofilm regeneration [34]. Together, the above experimental results suggest that TBF not only removes biofilms but also inhibits biofilm regeneration (Figure 5G).

### 2.6 Antibacterial Mechanism of TBF

**FIGURE 6:**
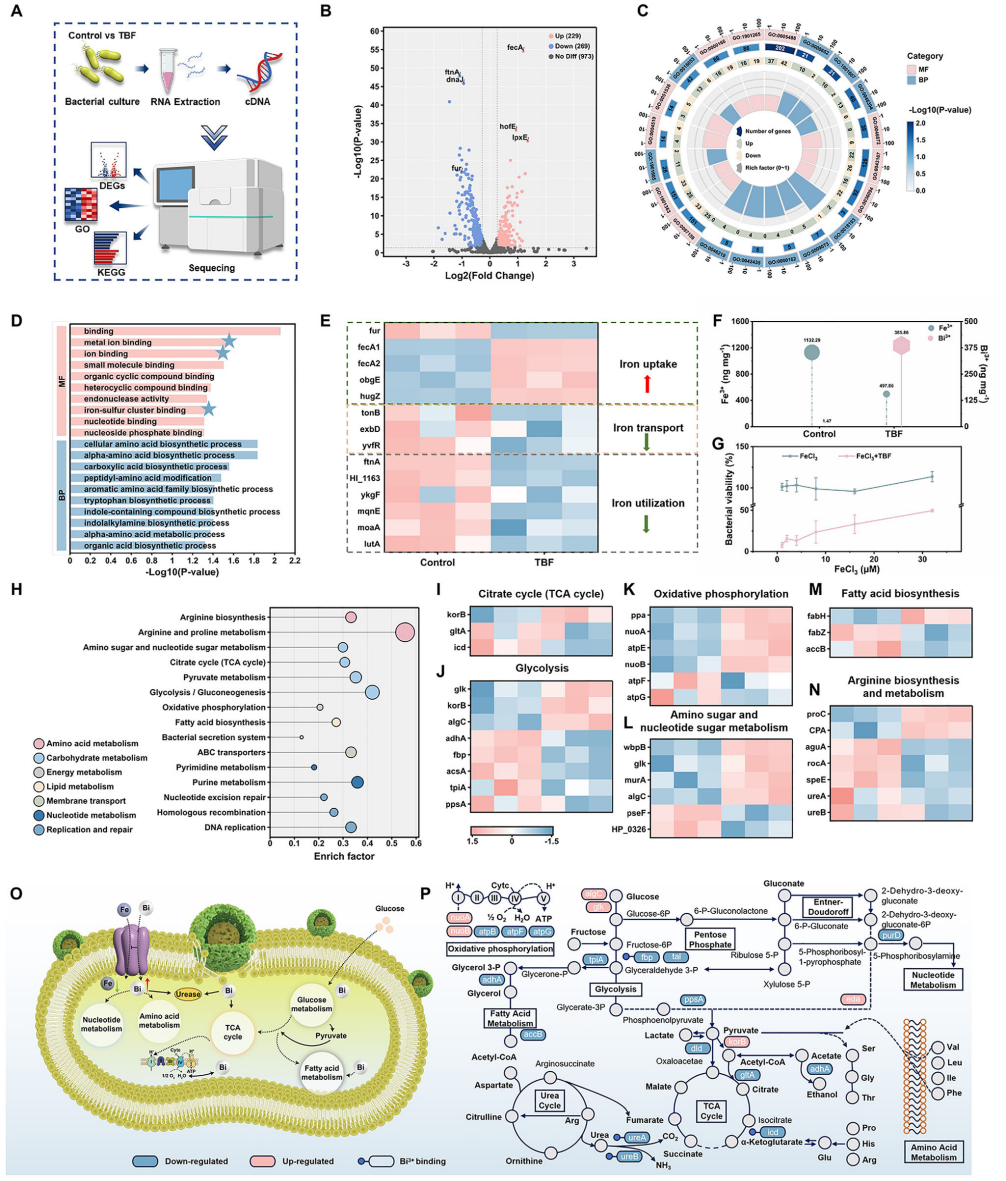
Transcriptomic analysis of *H. pylori* treated with TBF. (A) The *H. pylori* RNA sequencing workflow. (B) Volcano plot of DEGs. (C) GO pathway enrichment of DEGs. (D) TOP 10 pathways related to molecular functions and biological processes from GO enrichment analysis. (E) Heatmap of DEGs involved in Fe^3+^ uptake, transport and utilization. (F) Content of Fe^3+^ and Bi^3+^ in *H. pylori* after TBF treatment. (G) Bacterial viability after TBF treatment with and without the addition of Fe^3+^. (*n* = 3 biological replicates) (H) KEGG pathway enrichment of DEGs. Heatmap of DEGs involved in (I) TCA cycle, (J) glycolysis, (K) oxidative phosphorylation, (L) amino sugar and nucleotide sugar metabolism, (M) fatty acid biosynthesis, (N) arginine biosynthesis and metabolism. (O) Schematic diagram of pathways involved in TBF treatment of *H. pylori*. (P) Illustration of gene changes in bacterial nutrient metabolism pathways after treatment.

To further investigate how TBF exerts its bactericidal effect upon bacterial contact and elucidate its bactericidal mechanism, RNA was extracted from *H. pylori* with and without TBF treatment, followed by comparative transcriptomic analysis at the RNA level (Figure 6A). As shown in the differential expression gene (DEG) volcano plot, a total of 498 DEGs were identified in TBF‐treated *H. pylori* compared with untreated *H. pylori* (Control), including 229 up‐regulated genes and 269 down‐regulated genes (Figure 6B). Gene Ontology (GO) analysis of these DEGs focused on molecular functions, cellular components, and biological processes, revealing that TBF‐induced gene changes were mainly associated with molecular functions and biological processes (Figure 6C). Among these, metal ion binding, ion binding, and iron‐sulfur cluster binding were significantly affected (Figure 6D).

Subsequently, further analysis of the DEGs in the GO‐enriched pathways revealed that genes associated with iron uptake (*fecA*, *obgE*, *hugZ*) were upregulated, while those related to iron transport (*tonB*, *exbD*, *yvfR*) and utilization (*ftnA*, *HI_1163*, *ykgF*, *mqnE*, *moaA*, *lutA*) were downregulated (Figure 6E). Meanwhile, ICP‐MS detection results showed decreased intracellular Fe^3+^ concentration and increased Bi^3+^ concentration in the bacteria (Figure 6F). Furthermore, the antibacterial activity of TBF decreased after supplementing Fe^3+^ into the co‐culture system (Figure 6G). These results indicate that upon contact with *H. pylori*, TBF releases Bi^3+^, which competes with Fe^3+^ and disrupts the bacterium's iron uptake system. This interference with the normal uptake and utilization of Fe^3+^ leads to intracellular iron deficiency and bismuth overload in *H. pylori*, further confirming the nutritional trap strategy we proposed.

Fe^3+^ is an essential cofactor for various key enzymes involved in the metabolic activities of *H. pylori*, and iron deficiency will further lead to bacterial metabolic disorders [35, 36]. KEGG enrichment results showed that the gene level changes induced by TBF treatment affected multiple metabolism‐related pathways, including carbohydrate metabolism (glycolysis, pentose phosphate pathway, TCA cycle), lipid metabolism (fatty acid biosynthesis), and protein metabolism (arginine biosynthesis, arginine and proline metabolism) (Figure 6H). As shown in Figure 6I–K, TCA cycle‐related genes (*gltA*, *icd*), glycolysis‐related genes (*adhA*, *fbp*, *acs*, *tpiA*, *ppsA*), and oxidative phosphorylation‐related genes (*atpF*, *atpG*) were down‐regulated. These changes led to a decrease in ATP within the bacteria and a lack of energy supply for bacterial growth and reproduction. Coupled with the down‐regulation of genes related to amino sugar as well as nucleotide sugar metabolism and fatty acid biosynthesis, the raw materials for synthesizing cell membranes were reduced, and all these changes would lead to the lack of integrity of bacterial cell membranes and accelerate bacterial death (Figure 6L,M). Meanwhile, arginine biosynthesis, which is associated with biofilm formation and detachment, was also affected (Figure 6N). To further verify the alterations of relevant genes, we performed qPCR to detect the expression levels of representative genes, and the obtained results were consistent with transcriptomic data (Figure S11). In addition, the pathogenicity of *H. pylori* was suppressed after TBF treatment, and virulence factor‐associated genes (urea protein and cytotoxin‐associated genes) were also decreased (Figure S12).

Taken together, TBF exerts bactericidal effects on *H. pylori* by interfering with iron homeostasis and inhibiting metabolism. TBF captured the bacterial surface by target binding to the BabA protein on the surface of *H. pylori*, disrupting the bacterial cell membrane and releasing Bi^3+^. The intake of Bi^3+^ interferes with the normal regulation of Fe^3+^ in bacteria, breaking the iron homeostasis in bacteria, further decreasing the tolerance of bacteria to the environment, and disrupting the redox balance in bacteria (Figure 6O). Additionally, reduced metabolism of key nutrients impairs the bacteria's ability to maintain normal physiological activities, ultimately to death (Figure 6P).

### 2.7 In Vivo Evaluation of Antibacterial Activity

**FIGURE 7:**
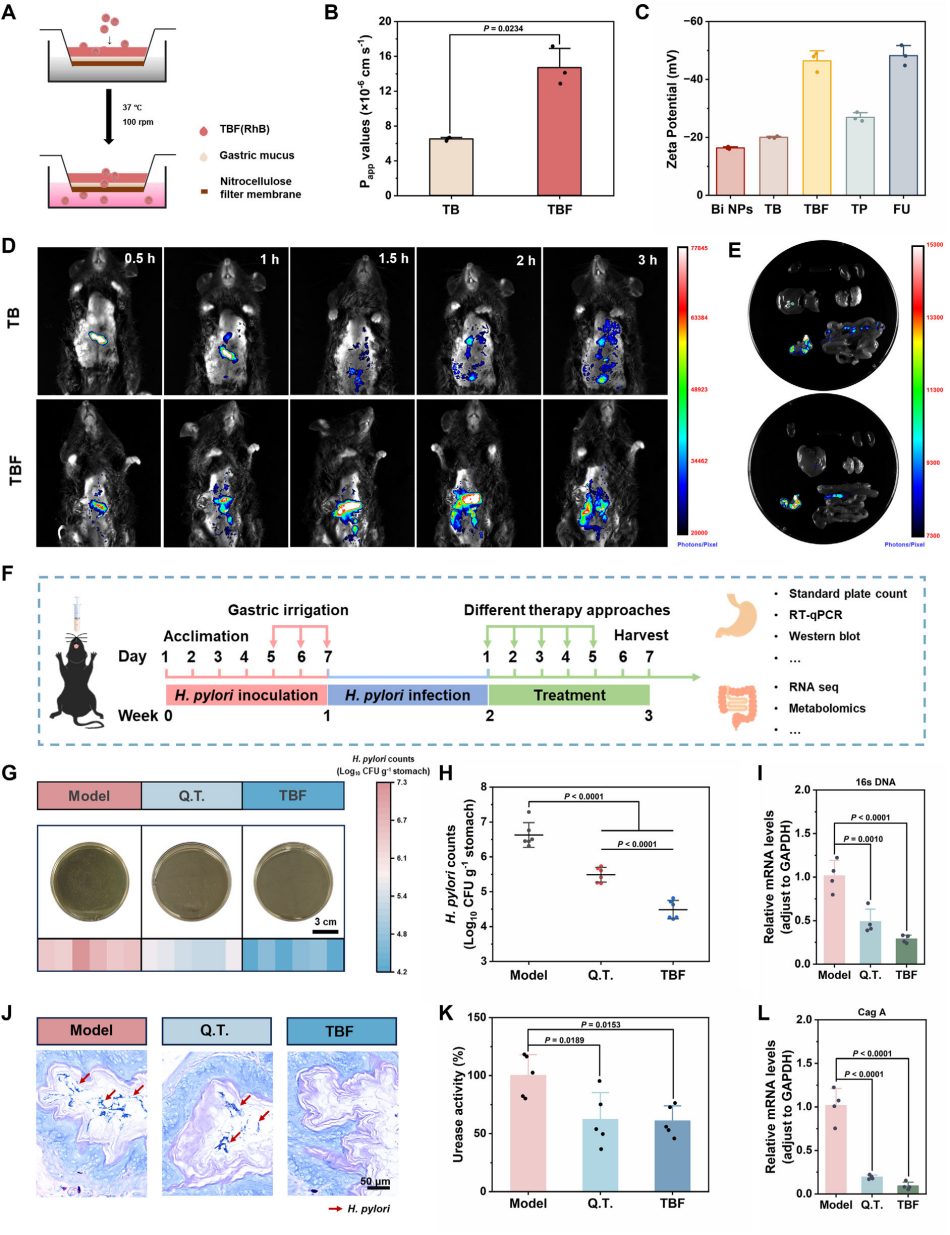
*H. pylori* infection therapy of TBF in vivo. (A) Schematic representative of the mucosal penetration test. (B) Mucus P_app_ values of TB and TBF. (*n* = 3 biological replicates, two‐sided Student's *t*‐tests) (C) Zeta potential of different nanodrugs. (*n* = 3 biological replicates) (D) Fluorescence images of mice. (E) Fluorescence images of mice's gastrointestinal tract and major organs at 3 h after nanodrug gavage. (F) Illustration of the mouse infection model establishment and treatment. (G) Representative photos of plates coated with mouse gastric tissue homogenates. (H) Statistics on the results of plate counting. (*n* = 6 biological replicates, one‐way ANOVA followed by post‐hoc Tukey's test) (I) The gene expression of *H. pylori* 16s DNA. (*n* = 4 biological replicates, one‐way ANOVA followed by post‐hoc Tukey's test) (J) Representative images of gastric tissue Giemsa staining. Red arrow: *H. pylori*. (K) Urease activity in gastric tissues after different therapies. (*n* = 5 biological replicates, one‐way ANOVA followed by post‐hoc Tukey's test) (L) The gene expression of Cag A. (*n* = 4 biological replicates, one‐way ANOVA followed by post‐hoc Tukey's test).

As *H. pylori* colonizes the area between the gastric submucosa and gastric epithelial cells, the mucus barrier in this region prevents drugs from reaching the infected site and reduces their effectiveness, making it necessary for effective drug delivery to overcome this obstacle [37]. In general, electrically neutral or negative substances are more likely to cross the gastric mucosa [38]. Herein, the coating of fucoidan was designed to increase the penetrability of the nanodrug. First, the penetration ability was verified in vitro by transwell assay (Figure 7A). As shown in Figure 7B, the penetration ability of TBF was significantly enhanced after encapsulating fucoidan. This may be due to the change in hydrophilicity and charge of TBF after encapsulation of fucoidan, giving it a stronger gastric mucosal penetration ability (Figure 7C). The ability of TBF to be retained in the stomach was subsequently verified. Generally, the time required for gastric emptying in mice was 0.5–3 h [39, 40]. After wrapping fucoidan, TBF slowly accumulated in the stomach with prolonged retention time (Figure 7D).

Compared with the TB group without wrapped fucoidan, more TBF stayed in the stomach, and only a few entered the intestine after 3 h of gavage administration (Figure 7E). Ion release assays revealed that TBF continuously released Bi^3+^ under simulated gastric peristalsis (Figure S13A), with significantly higher Bi^3+^ release in acidic simulated gastric fluid (SGF) than in neutral environments (Figure S13B). Further analysis demonstrated that SGF pretreatment did not impair the antibacterial efficacy of TBF (Figure S13C), and TBF exhibited enhanced anti‑*H. pylori* activity under acidic conditions compared with neutral environments (Figure S13D). These results suggest that TBF has the potential for oral delivery to the stomach for the treatment of *H. pylori* infection, and all these properties would be favorable to enhance the efficacy of TBF in vivo.

Subsequently, a high‐virulence *H. pylori* NSH57‐infected mouse model was established to study the antibacterial activity of TBF in vivo. As shown in Figure 7F, C57BL/6J mice were given 3 × 10^8^ CFU mL^−1^ *H. pylori* for three times to establish the infection model. The results of hematoxylin and eosin (H&E) staining showed colonization of *H. pylori*, as well as a partial inflammatory infiltrate (Figure S14). Subsequently, the urease activity test verified that the mouse stomach was colonized with *H. pylori*. (Figure S15). Together, these results indicated the successful establishment of a mouse gastritis model of *H. pylori* infection.

The in vivo therapeutic efficacy of TBF was validated after the model was established, and clinical quadruple therapy drugs were selected as the control group to compare the treatment effect of TBF. *H. pylori* colonization was assessed by the plate count method and quantitative polymerase chain reaction (qPCR). The results showed a decrease in bacterial load of at least two orders of magnitude in the TBF group, which was superior to the quadruple therapy (Q.T.) group (Figure 7G–I) and the single component group (Figure S16). And as visualized by the results of the Giemsa staining, there was a significant reduction in *H. pylori* colonization of the stomach (Figure 7J).

After colonizing the stomach, *H. pylori* secretes a variety of virulence factors, such as urease and Cag A, which cause damage to stomach tissue. Therefore, we compared the urease activity (Figure 7K) and measured the expression level of Cag A gene in gastric tissues after treatment by qPCR (Figure 7L). Both urease activity and Cag A expression were markedly suppressed upon TBF intervention. Together, these results suggested that TBF reduced *H. pylori* colonization and decreased the production of bacterial virulence factors.

Notably, the therapeutic outcomes in the TBF group were generally superior to those in the Q.T. group. We speculate that such efficacy differences are attributed to TBF's superior therapeutic effect against drug‐resistant bacteria and its prolonged retention in the stomach. TBF adheres to the surface of *H. pylori* and exerts bactericidal activity by disrupting bacterial iron homeostasis. This property confers TBF stronger bactericidal potency against drug‐resistant strains compared with quadruple therapy. In addition, TBF exhibits prolonged gastric retention, enabling sustained local accumulation in stomach tissues and extended therapeutic action time, thereby amplifying its in vivo antibacterial performance.

### 2.8 Recovery of Damaged Tissue

**FIGURE 8:**
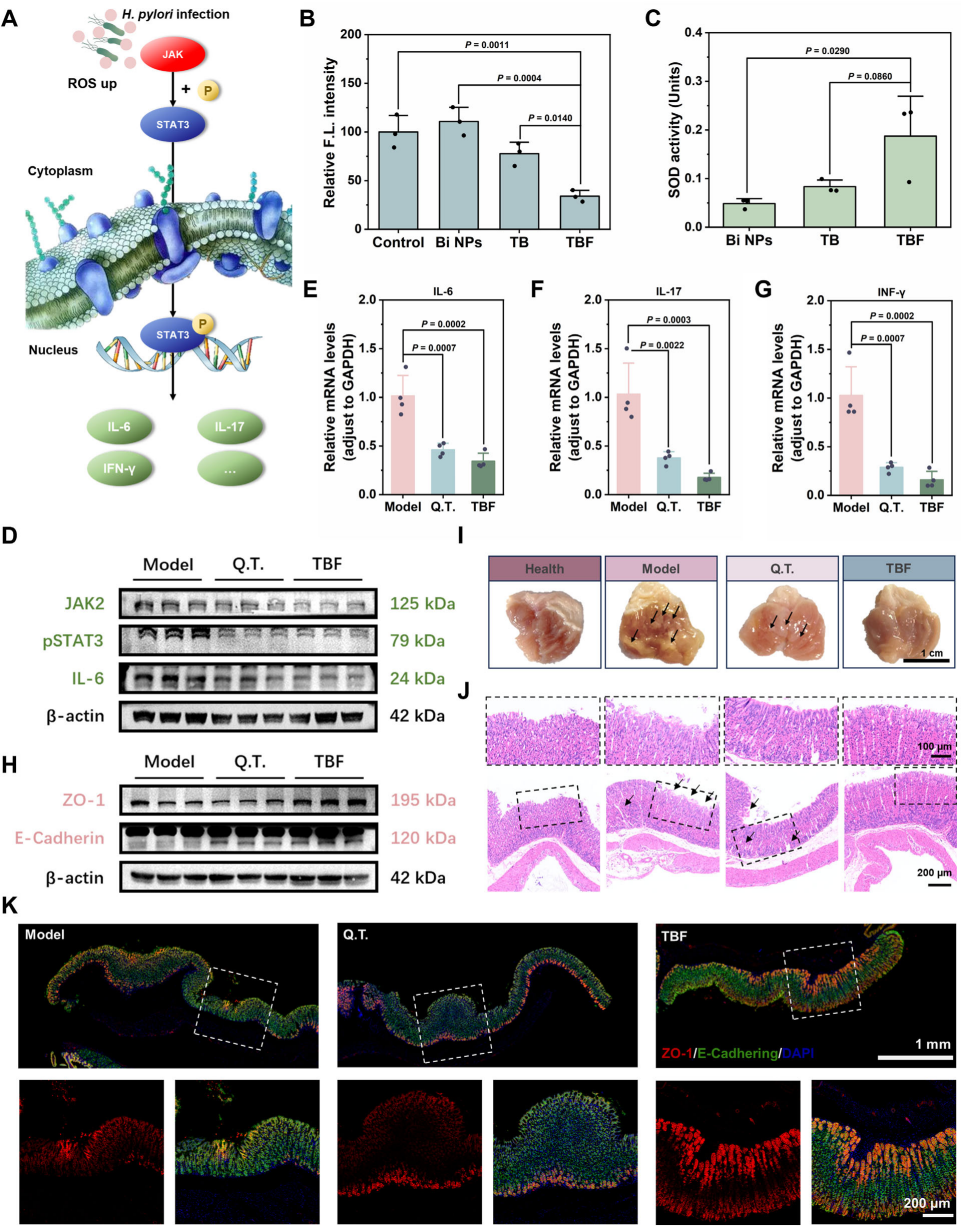
Inflammation relief and damaged tissue protection of TBF. (A) Schematic diagram of activation of the JAK/STAT pathway by *H. pylori* infection. (B) Fluorescence intensity of the system after different treatments was detected using the DCFH‐DA probe. (*n* = 3 biological replicates, one‐way ANOVA followed by post‐hoc Tukey's test) (C) SOD enzyme activity of different nanodrugs. (*n* = 3 biological replicates, one‐way ANOVA followed by post‐hoc Tukey's test) (D) The expression of JAK2, pSTAT3, and IL‐6. The levels of pro‐inflammatory factors (E) IL‐6, (F) IL‐17, and (G) IFN‐𝛾 in mice stomach. (*n* = 3 biological replicates, one‐way ANOVA followed by post‐hoc Tukey's test) (H) The expression of ZO‐1 and E‐Cadherin. Representative (I) photographs and (J) H&E‐stained images of gastric tissues before and after treatment. Black frame: representative area of tissue damage. Black arrow: gastric mucosal and acinar defect. (K) Representative images of immunofluorescence. White frame: representative area of tissue damage. Red area: ZO‐1. Green area: E‐Cadherin. Blue area: DAPI.

The Janus kinase‐signal transducer and activator of transcription (JAK‐STAT) signaling pathway, which mediates inflammatory responses and immunoregulatory processes, has been shown to play a critical role in gastric cancer tumorigenesis [41]. *H. pylori* infection expresses virulence factors and upregulates reactive oxygen species (ROS) levels, which stimulate and activate the JAK/STAT pathway and subsequently regulate the expression of pro‐inflammatory factors, leading to inflammation and even cancer at the site of infection (Figure 8A) [42, 43]. The results of in vitro experiments showed that the H_2_O_2_ scavenging ability of the nanodrug was gradually enhanced by the modification of polyphenols and fucoidan (Figure 8B), and this ability showed a concentration‐dependent pattern (Figure S17). In addition, TBF possessed some SOD activity (Figure 8C). This all contributes to the ROS scavenging by TBF at the infection site. Thus, TBF could remove *H. pylori* and reduce the production of virulence factors on the one hand, and remove ROS on the other hand, which would in turn prevent the activation of the JAK/STAT pathway. The expression of key proteins of the JAK/STAT pathway was subsequently detected by western blot. As shown in Figure 8D, the protein expression of JAK2 and pSTAT3 was significantly decreased after TBF treatment. Subsequently, the expression of downstream inflammatory factors was examined by qPCR, and a significant decrease in the expression of pro‐inflammatory factors IL‐6, IL‐17, and INF‐γ was also detected, indicating that inflammation was cleared (Figure 8E–G).

Gastric inflammation leads to damage of gastric tissues, and the detection of ZO‐1 and E‐Cadherin proteins revealed that the expression of these two proteins was significantly elevated after treatment by TBF (Figure 8H). Notably, gastric tissue from *H. pylori*‐infected mice exhibited obvious whitening and ulceration. Importantly, quadruple therapy failed to mitigate these pathological manifestations, as edema and inflammation remained prominent in the treated mice. The TBF group, on the other hand, had an intact surface that was close to normal gastric tissue (Figure 8I). Gastric tissue recovery was further assessed by H&E staining and immunofluorescence staining. Comparison revealed that the gastric mucosa of the TBF group was basically normal in organization and structure, and the epithelial cells of the gastric mucosa were neatly arranged without necrosis and detachment, and there were few lymphocyte aggregates. In contrast, obvious inflammatory cell infiltration was seen in the model group and Q.T. group, with aberrant gastric histology and disorganized glandular architecture (Figure 8J). Meanwhile, as shown in Figure 8K, the expression of tight junction protein in the epithelial cells of the TBF group was significantly higher than that of the other two groups and tended to be normalized (Figure S18), which was consistent with the results of the western blot. Together, these results suggest that TBF relieves inflammation and normalizes damaged tissue. It was superior to the clinical quadruple therapy group in this regard.

### 2.9 Regulation of Intestinal Flora

**FIGURE 9:**
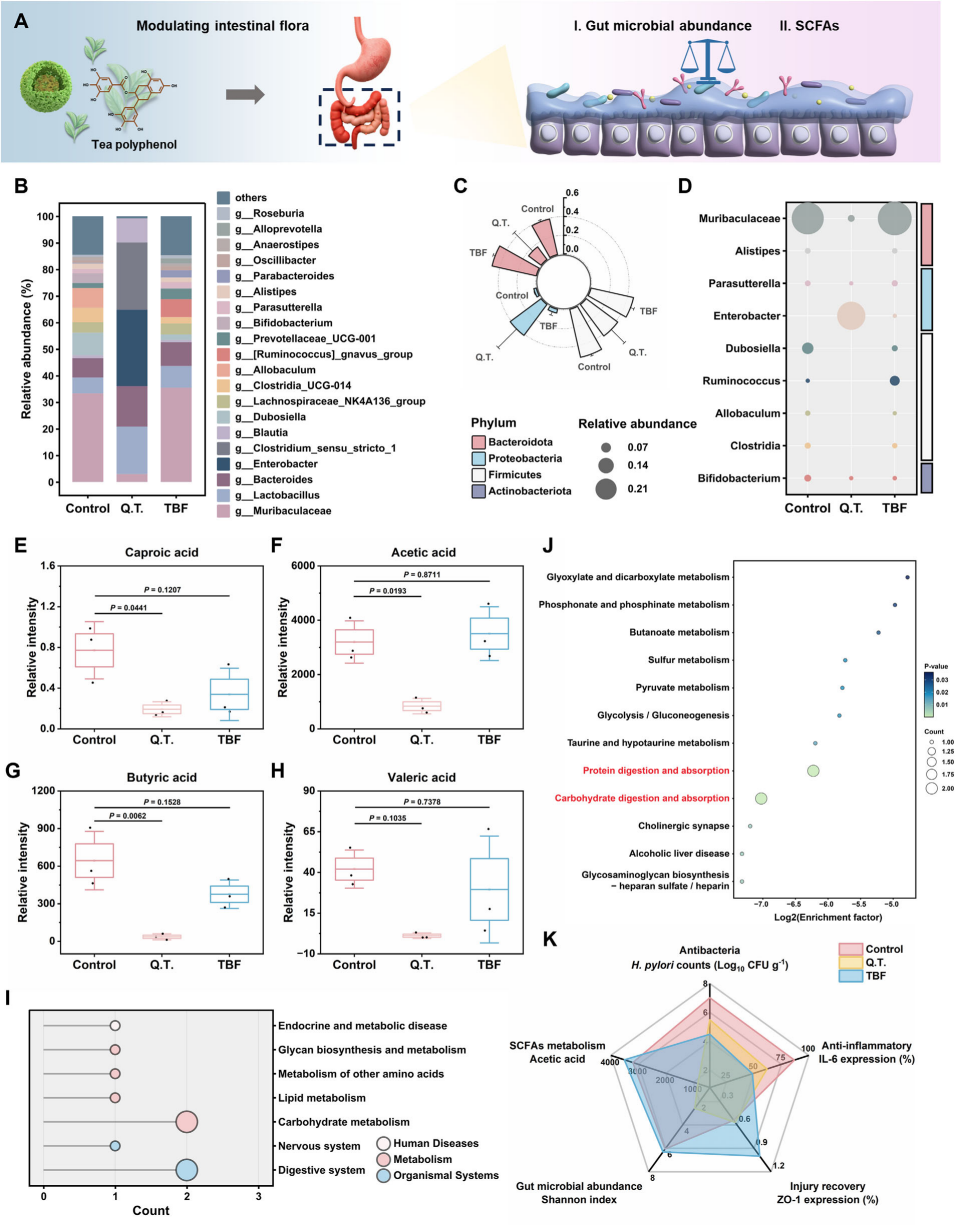
In vivo biosafety analysis of TBF. (A) Schematic representation of the regulation of intestinal flora and metabolism by TBF. (B) Relative abundance of gene levels in the mice's intestinal flora. (C) Relative abundance of *Proteobacteria*, *Firmicutes*, and *Bacteroidota*. (D) Differentially expressing strains at the gene level. Relative intensities of (E) caproic acid, (F) acetic acid, (G) butyric acid, and (H) valeric acid measured by targeted metabolomics. (*n* = 3 biological replicates, one‐way ANOVA followed by post‐hoc Tukey's test) (I) Lollipop chart for the KEGG pathway of differential metabolites. (J) Scatter plot for KEGG enrichment analysis of differential metabolites. (K) Radar plots of in vivo treatment effects.

One of the drawbacks of the existing antibiotic therapy is that it will damage the intestinal flora, and it has been reported that the intestinal flora of patients after quadruple therapy is still in a state of disorganization even after 1 year, which will affect the normal functioning of the body [44]. Considering this drawback, we incorporated polyphenols, a natural compound, into the design of TBF. Polyphenols have the potential to improve the composition and diversity of the intestinal flora and further modulate intestinal metabolites such as short‐chain fatty acids (SCFAs) (Figure 9A) [45, 46]. The effect of TBF on several probiotic bacteria was first verified in vitro. As shown in (Figure S19), lower concentrations of TBF showed a killing effect on pathogenic bacteria. However, for probiotics, even at a much higher concentration of 200 µg mL^−1^ (ten times more than the MIC of *H. pylori*), there was still no significant effect on their survival rate. Further analysis demonstrated that this selective killing effect was related to the addition of polyphenols (Figure S20). We also compared the intracellular ion concentration changes in probiotics and *H. pylori* after TBF administration. As shown in Figure S21, both strains exhibited similar trends in intracellular ion alterations. However, the lower iron dependency and higher bismuth tolerance of probiotics ensured their survival, in contrast to the lethal iron deficiency and bismuth overload induced in *H. pylori* [8, 10, 47, 48, 49]. This differential response underpins the selective antibacterial activity of TBF, preserving the gut microbiota while eradicating the pathogen.

Subsequently, we analyzed the intestinal flora of the treated mice by 16S rRNA gene‐sequencing. Sequencing results showed that the composition of the intestinal flora in the TBF group was very close to that of normal mice, whereas the intestinal flora in the Q.T. group was obviously damaged by the drug and had a more homogeneous flora composition (Figure 9B). The Venn diagram of the ASV analysis showed that the composition of the intestinal flora in the TBF group overlapped with most of that of the healthy control group, whereas there was only a small portion of crossover in the Q.T. group (Figure S22).

Further generalization and analysis of the flora composition between the groups revealed that the changes in bacterial abundance mainly occurred in *Proteobacteria*, *Firmicutes*, and *Bacteroidota* (Figure 9C). Among them, the *Proteobacteria*, which consists mostly of pathogenic bacteria and is involved in the development of human diseases, was significantly up‐regulated after antibiotic treatment, whereas the abundance of the *Firmicutes* and *Bacteroidota*, a major contributor of SCFAs that is mostly composed of probiotic bacteria and associated with the metabolism of a wide range of nutrients, was down‐regulated in response to antibiotics. In contrast, these bacterial communities were still maintained at normal levels after TBF treatment. Subsequently, the differentially expressed strains were analyzed at the genus level. As shown in Figure 9D, SCFAs producers *Muribaculaceae*, *Ruminococcus*, and *Clostridia*, which almost disappeared in the Q.T. group, were restored by TBF treatment. Meanwhile, the abundance of *Enterobacter*, a pathogenic bacterium that increased significantly after antibiotic treatment, remained at normal levels. Together, these results suggested that TBF treatment did not affect the composition and diversity of the intestinal flora compared to antibiotic therapy.

Intestinal flora balance is closely related to nutrient metabolism and disease occurrence. Thus, we next performed a targeted metabolomic analysis of SCFAs, a major component of intestinal metabolites. Although the intestinal metabolism of caproic acid, acetic acid, and butyric acid was significantly decreased in mice treated with quadruple therapy (Figures 9E–H), the metabolism of seven short‐chain fatty acids still remained at normal levels in the TBF group (Figure S23), which was consistent with the changes in the intestinal flora. KEGG enrichment results showed that changes in SCFAs metabolism induced by treatment with antibiotics affect human diseases, metabolism, and organismal systems (Figure 9I). It mainly affected protein metabolism and glucose metabolism, which in turn induce a range of diseases (Figure 9J). In contrast, TBF did not interfere with the metabolism of SCFAs, triggering the risk of disease. In addition, the body weights of mice remained stable without significant fluctuations throughout the entire experimental period (Figure S24), and no noticeable pathological lesions were observed in major organs post‑treatment (Figure S25). Together, the above experimental results demonstrate that TBF not only effectively eradicates bacteria, alleviates inflammation, and protects the damaged gastric mucosa, but also preserves the homeostasis of intestinal flora and metabolites in a mouse model of *H. pylori*‐infected gastritis. Furthermore, its therapeutic efficacy is, to a certain extent, superior to that of quadruple therapy (Figure 9K).

## 3 Conclusion

*H. pylori*, classified as a Group I carcinogen by the International Agency for Research on Cancer (IARC), infects more than half of the global population. Current antibiotic‐based first‐line therapies face prominent challenges including bacterial resistance, biofilm obstruction, and intestinal flora disruption, creating an urgent clinical demand for safer and more effective antibiotic‐alternative therapies. Addressing this unmet need, the bismuth‐based nanodrug TBF, designed based on the nutritional trap strategy, offers a targeted therapeutic approach for *H. pylori* infection. Validated by experimental data, TBF's multifaceted biological activities address key challenges of conventional antibiotic therapies. It efficiently eradicates *H. pylori*, including clinically isolated multidrug‐resistant strains, exhibits potent activity against *H. pylori* biofilms, and scarcely induces bacterial resistance. Notably, the polyphenol component endows TBF with selective bactericidal capacity—eliminating pathogenic *H. pylori* while preserving probiotics, thereby maintaining the balance of intestinal flora and metabolites post‐treatment. This advantage is particularly valuable, as gut microbiota disruption is a major drawback of traditional antibiotic regimens that often leads to adverse gastrointestinal outcomes.

The therapeutic efficacy of TBF stems from its well‐orchestrated mechanism of action. Endowed with a negative charge, TBF penetrates the subgastric mucosa and specifically targets *H. pylori* via fucoidan's binding to the bacterial BabA protein. Subsequent release of Bi^3+^ mimics Fe^3+^ to deceive the bacterium's iron‐uptake system, forming a nutritional trap that disrupts iron homeostasis by inducing intracellular bismuth overload and iron deficiency. Transcriptomic analysis further corroborated this mechanism by revealing perturbed expression of *H. pylori* genes associated with iron uptake. Concomitantly, key bacterial metabolic pathways, including the TCA cycle, glycolysis, amino acid metabolism, and fatty acid biosynthesis, are impaired, ultimately leading to bacterial death. In vivo studies confirmed that TBF reduces *H. pylori* gastric colonization by nearly three orders of magnitude, while alleviating gastric inflammation and tissue damage, validating its translational potential. Considering potential tissue accumulation risks, future work will conduct comprehensive druggability evaluations of TBF, including pharmacokinetic profiles, long‑term biosafety and toxicological assessments, to systematically evaluate its clinical safety.

Overall, TBF's core mechanism—mediating a nutritional trap to disrupt *H. pylori* iron homeostasis—addresses the unmet need for antibiotic‐alternative therapies. Its selective bactericidal activity and gut microbiota‐preserving property highlight its potential to reshape *H. pylori* treatment paradigms, offering a valuable option for patients with antibiotic‐resistant infections or those intolerant to conventional regimens. Beyond *H. pylori*, the nutritional trap strategy may serve as a template for developing targeted therapies against other iron‐dependent pathogens, expanding the scope of antimicrobial development and addressing the global challenge of antimicrobial resistance.

## 4 Experimental Section

### 4.1 Ethics Approval Statement

All experimental procedures involving animals were conducted in accordance with the guidelines for the care and use of laboratory animals and were approved by the Institutional Animal Care and Use Committee (IACUC) of China Pharmaceutical University (YSL‐202505051).

### 4.2 Materials

Bismuth nitrate anhydrous (Bi(NO_3_)_3_·5H_2_O), Tea polyphenols, Fucoidan, Dimercaprol (BAL), Crystal violet, Chrome Azurol S (CAS), Rhodamine B, and Urea were purchased from Shanghai Macklin Biochemical Co., Ltd (Shanghai, China). Glycine, Polyvinylpyrrolidone (PVP‐K10), Sodium hydroxide (NaOH), Sodium borohydride (NaBH_4_), Acetic acid, and Dichlorofluorescin Diacetate (DCFH‐DA) were purchased from Shanghai Aladdin Biochemical Technology Co., Ltd (Shanghai, China). Brucella Broth, *H. pylori* solid medium, Mueller‐Hinton medium, and MRS Broth were provided by Qingdao Haibo Biological Co., Ltd (Qingdao, China). Defibrinated Sheep Blood was purchased from Changde BKMAM Biotechnology Co., Ltd (Hunan, China). SYTO‐9, SYTO X, and Propidium Iodide (PI) were obtained from Nanjing Keygen Biotech Co., Ltd (Jiangsu, China).

### 4.3 Synthesis of Bi NPs

Bi NPs were prepared by the chemical reduction method according to previous work with some modification [18]. Briefly, Bi(NO_3_)_3_·5H_2_O (146.2 µg, 15 mm) was added to the pre‐warmed glycine solution (20 mL, 1 m). Then, NaOH was added dropwise to raise the solution pH to 9, and the color of this solution turned white during this process. After 3 min stirring, BAL (75 µL, 8.1 m) and PVP‐K10 (3 mL, 3 mm) were added to the mixture, rapidly turning the solution color to bright yellow. Finally, NaBH_4_ (7 mL, 1 m) was used to reduce Bi^3+^. The product was obtained by freeze‐drying after washing and stored at 4°C.

### 4.4 Synthesis of TB and TBF

Bi NPs and tea polyphenols were added to water at a mass ratio of 2:1, dispersed homogeneously, and then stirred at room‐temperature for 2 h. Subsequently, fucoidan equal to the mass of the Bi NPs was added, and stirring was continued at room‐temperature for 0.5 h. The impurities were removed by dialysis, and the powdered state of the TBFs was obtained by lyophilization.

### 4.5 Characterization of Material

TEM (FEI Tecnai T20, Thermofisher, United States) was used to characterize the morphology of TBF. The chemical composition of Bi NPs, TB, and TBF was characterized by FTIR (Vertex 70, Bruker, United States) and UV–vis Spectrophotometer (UV 1800, Shimadzu, Japan). FTIR spectra were recorded using OPUS5.5 software for 4000–500 cm^−1^ with a resolution of 4 cm^−1^. XRD (AG‐DSC823e, Mettler Toledo, Switzerland) was employed to analyze the crystalline form of TBF. XPS (ESCALAB 250XI, Thermofisher, United States) was performed for analyzing the chemical valences of TBF. DLS and zeta potential was performed for basic characteristics and stability of TBF by Nanometrics (Zetasizer Nano, Malvern, United Kingdom).

### 4.6 *H. pylori* Cultivation

The *H. pylori* strain ATCC 26695 and clinical *H. pylori* strains were generously provided by the Key Laboratory of Tumorigenesis and Intervention in Jiangsu Province. These bacteria were cultured on Columbia blood plates, which were supplemented with 10% defibrinated sheep blood. The cultivation was carried out in a three‐gas incubator maintained at 37°C, with an atmosphere of 5% O_2_, 10% CO_2_, and 85% N_2_, for a period of 72–96 h.

### 4.7 CAS Assay

*H. pylori* in the logarithmic growth phase was scraped, placed in Brucella Broth, and resuspended to a bacterial suspension of 1 ×10^7^ CFU mL^−1^. It was co‐cultured with Bi NPs at the MIC concentration for 12 h. The mixture was centrifuged at 10 000 r·min^−1^ for 10 min. Then, 3 mL of the supernatant was taken and mixed with an equal volume of 2 × CAS detection solution. The mixture was allowed to stand in the dark for 30 min, after which the absorbance at 630 nm was measured. The blank CAS liquid medium without bacterial inoculation was used as the negative control group. The relative siderophore production was calculated using the equation provided below:

$\mathrm{Relative}\;\mathrm{siderophore}\;\mathrm{production}(\%)\;=\frac{OD_{630r}-OD_{630s}}{OD_{630r}}\;\times 100\%$

Where OD_630r_ is the absorbance at 630 nm of the negative control group, OD_630s_ is the absorbance of the experimental sample at 630 nm.

### 4.8 Bacterial Viability Assay

*H. pylori* in the logarithmic growth phase was collected and prepared into a bacterial suspension with a concentration of 1 ×10^7^ CFU mL^−1^. The bacterial suspension was co‐incubated with nanodrugs (Bi NPs, TB, TBF) at their respective MIC concentrations, and FeCl_3_ at concentrations ranging from 0 to 32 µm was added to the culture system simultaneously. After 72 h, the absorbance of the co‑culture system at 600 nm was detected. The bacterial viability was calculated using the equation provided below:

$\mathrm{Bacterial}\;\mathrm{viability}(\%)\;=\frac{OD_{600s}-OD_{600b}}{OD_{600c}-OD_{600b}}\;\times 100\%$

Where OD_600b_ is the absorbance at 600 nm of the blank culture medium without bacteria, OD_600s_ is the absorbance of the drug‑treated group at 600 nm, OD_600c_ is the absorbance of the control group at 600 nm.

### 4.9 ICP‐MS Detection

Bacterial suspensions (5 × 10^8^ CFU mL^−1^) treated with different nanodrugs (50 µg mL^−1^) were collected, washed three times with sterile water to remove residual drugs, and the bacterial cells were harvested. Then, nitric acid (5% v/v, 5 mL) was added for digestion. Subsequently, the remaining nitric acid was evaporated at 160°C, and the solution was diluted to 25 mL with Grade I water as specified in GB/T 6682. Rhenium (Re) and arsenic (As) were selected as internal standards, and the concentrations of Bi^3+^ and Fe^3+^ were determined by ICP‐MS (8900 ICP‐MS Triple Quad, Agilent, United States).

### 4.10 In Vitro Cytotoxicity

To confirm the biocompatibility of TBF, the cytotoxicity of TBF was initially examined. GES‐1 (RRID: CVCL_EQ22), sourced from the culture collection of the Chinese Academy of Sciences (Shanghai, China), was employed to assess the cytotoxicity of TBF through the CCK8 assay. These cells were initially cultivated in DMEM medium supplemented with 10% fetal bovine serum, penicillin at a concentration of 100 U mL^−1^, and streptomycin at a concentration of 100 mg mL^−1^. Every 3 days, the cells were harvested by treating them with 0.25% trypsin solution for subculture, and then used for the cytotoxicity tests.

The CCK‐8 assay was performed using the reduction of WST‐8 to formazan as an indicator of cell viability. The cells were seeded in 96‐well plates at a density of 3 × 10^3^ cells mL^−1^ and allowed to attach and grow overnight. Subsequently, the original growth medium was replaced with DMEM solutions containing different concentrations of the nanodrugs (3.12, 6.25, 12.5, 25, 50, 100, 200 µg mL^−1^) and incubated for 24 h. After that, 10 µL of CCK‐8 solution was added to each well, and the plates were further incubated for 4 h at 37°C. Finally, the optical density values were measured at 450 nm. The positive control group represented the normal culture control group without the addition of nanodrugs, while the negative control group denoted the blank control group free of both cells and medicines. The cell viability was calculated using the equation provided below:

$\mathrm{Cell}\;\mathrm{viability}(\%)\;=\frac{OD_{450s}-OD_{450n}}{OD_{450p}-OD_{450n}}\;\times 100\%$

Where OD_450s_ is the absorbance of the experimental sample at 450 nm wavelength, OD_450n_ is the absorbance at 450 nm wavelength of the negative control group, and OD_450p_ is the absorbance at 450 nm wavelength of the positive control group.

### 4.11 Hemolysis Test

To assess the hemocompatibility of TBF, a hemolysis assay was conducted utilizing mouse erythrocytes. Fresh blood was collected from mice by sampling the blood from their eyeballs. Subsequently, the erythrocytes were separated through centrifugation at 1500 rpm for 1 min. After separation, the erythrocytes were washed three times with 0.9% physiological saline. Following the washing process, the precipitated erythrocytes were resuspended in 0.9% physiological saline to prepare a 2% erythrocyte suspension.

Concurrently, TBF solutions with varying concentrations (1.56, 3.12, 6.25, 12.5, 25, 50 µg mL^−1^) were prepared. Then, 500 µL erythrocyte suspension was co‐incubated with 50 µL TBF solutions at different concentrations in a water bath at 37°C for a duration of 3 h. After the co‐incubation period, the mixture was centrifuged once again at 1500 rpm. The supernatant was carefully collected, and its absorbance value was measured at a wavelength of 540 nm. For the positive and negative control groups, 50 µL of the nanodrugs were substituted with 50 µL of deionized water and physiological saline, respectively. The hemolysis rate was calculated using the equation provided below:

$\mathrm{Hemolysis}\;\mathrm{rate}(\%)\;=\frac{OD_{540s}-OD_{540n}}{OD_{540p}-OD_{540n}}\;\times 100\%$

Where OD_540s_ is the absorbance of the experimental sample at 540 nm wavelength, OD_540n_ is the absorbance at 540 nm wavelength of the negative control group, and OD_540p_ is the absorbance at 540 nm wavelength of the positive control group.

### 4.12 Molecular Docking

The crystal structure of the BabA protein (PDB ID: 4ZH0) was obtained from the Protein Data Bank database (http://www.rcsb.org/). The three‐dimensional structure of the fucoidan ligand was acquired from the PubChem database (http://pubchem.ncbi.nlm.nih.gov/). Protein and ligand preprocessing was completed by AutoDock Tools software. The grid box was set with central coordinates at X = 1.469, Y = −14.381, Z = 6.142 to fully cover the active binding pocket of BabA. Subsequently, semi‐flexible molecular docking was carried out via AutoDock Vina, where ligands were allowed to adjust conformations freely while the protein remained rigid. The docking results were evaluated according to the built‐in scoring function, and intermolecular interaction characteristics were further analyzed by Protein‐Ligand Interaction Profiler. Finally, all docking conformations were visualized by PyMOL software.

### 4.13 Adhesion Competitive Inhibition Assay

Log‑phase *H. pylori* were collected and prepared into a bacterial suspension at a concentration of 1 × 10^7^ CFU mL^−1^. The bacterial suspension was co‑incubated with fucoidan (25, 50, 100, 200 µg mL^−1^) for 3 h, followed by serial dilution and spreading onto Columbia blood agar plates. After incubation for 72 h in a tri‑gas incubator, colony‑forming units were counted. In parallel, *H. pylori* pre‑incubated with fucoidan (25, 50, 100, 200 µg mL^−1^) for 3 h were treated with 25 µg mL^−1^ TBF for 12 h. The treated bacterial suspensions were serially diluted and spread onto Columbia blood agar plates, and colony counts were determined after 72 h of incubation under microaerobic conditions.

### 4.14 Bacterial SEM Observations

The bactericidal performance and bacterial capture ability of different treatments were visualized via SEM (Sigma 360, Zeiss, Germany). Bacterial suspensions (5 × 10^8^ CFU mL^−1^) treated with TBF (50 µg mL^−1^) were collected, washed three times with sterile water to remove residual drugs. The bacterial cells were harvested and fixed with a 2.5 wt.% glutaraldehyde solution and dehydrated with a gradient of ethanol/water solution. Subsequently, the SEM images were acquired to observe the bacterial capture ability and their morphologies.

### 4.15 Validation of TBF Interactions with Bacteria

The adhesion of TBF to the bacterial surface and its entry into the bacteria was detected using flow cytometry (FACSAriaIII, Becton Dickinson, United States) and ODT techniques (HT‐2H, Tomocube, Korea). TBF (12.5 µg mL^−1^) was fluorescently labeled using Rhodamine B and subsequently co‐cultured with 1 × 10^8^ CFU mL^−1^ bacteria. Bacteria was taken at different time points, centrifuged, and washed three times. The bacterial fluorescence was detected using flow cytometry.

Besides, the ability of TBF to enter the interior of the bacteria was verified by ODT. Raw holograms from multi‑angle illumination were preprocessed by background subtraction and noise reduction. 2D phase maps of *H. pylori* were retrieved via digital holography. According to the Fourier diffraction theorem, phase data were mapped into the 3D Fourier space, followed by inverse Fourier transformation to reconstruct three‑dimensional refractive index tomograms. Post‑processing was performed to correct imaging artifacts, and analysis of bacterial structural changes was conducted [28].

### 4.16 MIC Test

MIC was determined according to CLSI (M45) standards. Briefly, agar dilution plates containing the antimicrobial agent were prepared first. Subsequently, log‑phase *H. pylori* were suspended in Brucella Broth and adjusted to a concentration of 1 × 10^7^ CFU mL^−1^. A volume of 1–2 µL of the bacterial suspension was spotted onto each drug‑containing agar plate using a multi‑point inoculator or micropipette. After incubation for 72 h in a tri‑gas incubator, MIC values were determined by visual inspection, defined as the lowest concentration at which no visible bacterial colonies were observed.

### 4.17 Resistance Studies

To assess if repeated administration of antibiotics or nanodrugs could lead to the development of bacterial resistance, a series of consecutive passages of bacterial growth inhibition assays were carried out [50, 51]. The *H. pylori* strain ATCC 26695 was selected as a representative strain, and clarithromycin was chosen as a typical antibiotic for this investigation. Determination of MIC was performed according to the above method. Bacterial cultures treated at 0.5 × MIC of a previous inhibition assay were subsequently adjusted the density to 1 × 10^7^ CFU mL^−1^ as the final bacterial inoculum for the next growth inhibition assay. Such cyclic inhibition assays were continued for over ten passages till the MIC values reached a plateau where they are steady for ≥ 5 consecutive assays.

### 4.18 Plate Counting Method

Agar plate counting was performed as the following steps. First, Bi NPs, TB and TBF with different concentrations (3.125, 6.25, 12.5, 25, 50, 100 µg mL^−1^) were added to 1 mL of bacterial solution (1 × 10^7^ CFU mL^−1^) and incubated in a three‐gas incubator for 12 h. The mixtures were further diluted and applied on co‐cultivated blood plates for 72 h to perform colony counting.

### 4.19 Live/Dead Staining Test

*H. pylori* (1 × 10^8^ CFU mL^−1^) in the logarithmic growth phase were incubated with different nanodrugs (25 µg mL^−1^) for 12 h. The mixture was then centrifuged, resuspended, and washed to collect the planktonic bacteria. The bacteria were then stained with SYTO 9 (2.5 µm) and propidium iodide (10 µg mL^−1^) at 37°C for 30 min. Once the staining was completed, the stained bacteria were observed and quantitatively analyzed using a fluorescence microscope (Ti2, Nikon, Japan) [52].

### 4.20 Inhibition of *H. pylori* Adhesion to GES‐1 Cells

The bacterial adhesion to cells was observed using the crystal violet staining method [37]. First, TBF (25 µg mL^−1^) was co‐cultured with GES‐1 cells for 1 h. Subsequently, *H. pylori* (1 × 10^6^ CFU mL^−1^) in the logarithmic growth phase was taken to infect the cells (MOI = 50:1) and continued to be cultured for 3 h. The medium was discarded, washed three times, and then fixed with a 10% methanol solution and stained with a 0.1% crystal violet staining solution. Finally, the bacterial adhesion to the cells was visualized under the inverted biological microscope (DSZ2000X, Reboot Optics, China).

### 4.21 Leakage of Bacterial Contents

To assess the leakage of cellular contents resulting from bacterial membrane disruption, the levels of K^+^, DNA, and proteins in the extracellular environment were measured following drug treatment. Bacteria were harvested from the bacterial solution by centrifugation at 5000 rpm for 5 min. The leakage level of DNA was determined using a microplate reader at a wavelength of 260 nm (SpectraMax i3x, Molecular Devices, United States). For the measurement of K^+^ (Nanjing Jiancheng Co., Ltd., Cat#C001‐2‐1) and protein (Nanjing Jiancheng Co., Ltd., CAT#KTD3001) leakage levels, specific assay kits were utilized, respectively.

### 4.22 DNA Damage Assay

Agarose gel electrophoresis was employed to evaluate the damage inflicted on bacterial genomic DNA. In the investigation of genomic DNA damage, the bacterial suspension (1 × 10^8^ CFU mL^−1^) was treated with different nanodrugs (Bi NPs, TB, and TBF, 25 µg mL^−1^) under microaerobic conditions for 12 h. Subsequently, the supernatant was removed through centrifugation at 5000 rpm for 5 min. Genomic DNA was then isolated using a genomic DNA extraction kit (Omega Biotechnology, Cat#D3350‐01). After extraction, the concentration of genomic DNA was measured using a microplate reader (SpectraMax i3x, Molecular Devices, United States) at a wavelength of 260 nm. Finally, 1% agarose gel electrophoresis with GelRed was utilized to detect the DNA cleavage products. The electrophoresis was carried out at 120 V for 30 min, and the results were analyzed with a gel imaging analysis system (4600SF, Tanon, China).

### 4.23 Motility Test

Motility testing was carried out following the method detailed by Suerbaum et al. [53]. Briefly, Brucella agar supplemented with 10% BSA was utilized, and two‐layered plates were prepared to assess the motility of the bacteria. The bottom layer of the plate contained 1.5% agar. After pouring this bottom layer onto the plates, a soft upper layer containing 0.4% agar and different nanodrugs were added. Subsequently, *H. pylori* (1 × 10^7^ CFU mL^−1^) in the logarithmic growth phase was inoculated onto the plates under microaerobic conditions for three days. The motility of bacteria is determined by observing the diffusion and growth of bacteria around the inoculation puncture line.

### 4.24 ATP Levels of Bacterial

*H. pylori* (1 × 10^8^ CFU mL^−1^) in the logarithmic growth phase were collected and co‐cultured with different nanodrugs (25 µg mL^−1^) respectively. Subsequently, the ATP level of the bacteria was determined by BacTiter‐Glo Microbial Cell Viability Assay (Promega Biotechnology, Cat#TB337).

### 4.25 Iron‑Rescue Assay

Log‑phase *H. pylori* (1 × 10^8^ CFU mL^−1^) were co‑incubated with 25 µg mL^−1^ TBF and serially diluted FeCl_3_ (8, 16, 32, and 64 µg mL^−1^). The mixtures were cultured in a tri‑gas incubator for 12 h. After centrifugation at 5000 rpm for 5 min, the supernatants were collected, and the protein and DNA leakage levels were determined following the method for intracellular content leakage measurement. The harvested bacterial pellets were stained with PI (10 µg mL^−1^) at 37°C for 30 min, and fluorescence intensity was measured at 535/617 nm (Ex/Em). Meanwhile, genomic DNA was extracted from treated bacteria and subjected to agarose gel electrophoresis.

### 4.26 Validation of Downstream Toxicity of DFX Against *H. pylori*

Log‑phase *H. pylori* (1 × 10^8^ CFU mL^−1^) were treated with deferasirox (DFX) at concentrations of 0.5, 1, 2, and 4 µm, and incubated in a tri‑gas incubator for 12 h. After centrifugation at 5000 rpm for 5 min, the supernatant and bacterial pellets were collected separately. The protein concentration in the supernatant was determined using the BCA method, while genomic DNA was extracted from the pellets and analyzed by agarose gel electrophoresis.

### 4.27 Biofilm Cultivation

*H. pylori* biofilm was cultivated in sterile 96‐well plates following a standard method [54]. First, *H. pylori* grown to logarithmic phase were scraped from blood plates, suspended in Brucella Broth, and centrifuged at 4000 rpm to adjust the density to 1 × 10^8^ CFU mL^−1^. Subsequently, 100 µL of the adjusted bacterial solution was added to each well of the 96‐well plates, while 1 mL of the same solution was added to confocal dishes. The plates and dishes were then incubated under microaerobic conditions for a period of 72–96 h. After this incubation, the Brucella Broth was carefully removed, and the cultures were gently rinsed three times with sterile saline.

### 4.28 eDNA Detection Assay

eDNA is a crucial constituent of the biofilm extracellular matrix. Therefore, SYTO X was employed to assess the removal of immature biofilms [55]. *H. pylori* biofilms were grown on a confocal dish as described above with a shorter culture time (24 h), and different nanodrugs (25 µg mL^−1^) were added then. After incubation for another 12 h, the biofilms were gently rinsed with a saline solution. Then, they were incubated in the dark with a 2 µm SYTO X solution for 30 min for staining. The stained biofilms were examined using a confocal laser scanning microscope (LSM900, Zeiss, Germany). The acquired images were initially processed with the ZEN imaging software (version: 3.9).

### 4.29 Biofilm Biomass Detection

To evaluate the efficacy of different treatments in eliminating mature biofilms, a crystal violet assay was initially conducted. After obtaining the mature biofilm, various nanodrugs were introduced into each well, and the incubation was prolonged for 12 h. Following the incubation, the culture medium was carefully removed. The biofilms were then gently rinsed with sterile saline to eliminate planktonic cells and stained with 1% (w/v) crystal violet solution for 30 min. After staining, 200 µL of 33% acetic acid was added. The biomass of the biofilms was quantified by measuring the absorbance of the acetic acid solutions at a wavelength of 570 nm.

To further confirm the removal of mature biofilms, the plate counting method was employed. After treatment, the remaining biofilm was resuspended in Brucella Broth. The suspension was then serially diluted and spread onto co‐cultivated blood plates. After a 72 h incubation period, the colonies on the plates were counted. Moreover, the quantities of biofilm polysaccharides and proteins were determined using the sulfate‐phenol method and the BCA method, respectively [56].

### 4.30 Biofilm Formation Inhibition Evaluation

To evaluate the effects of nanodrugs on biofilm formation, the nanodrugs (25 µg mL^−1^) were added to 96‐well plates after the formation of mature biofilm, and the biofilm biomass was measured by crystal violet staining at 12, 24, 48, and 72 h, respectively.

### 4.31 RNA‐seq Analysis

1 × 10^8^ CFU mL^−1^ *H. pylori* in the logarithmic growth phase were collected and co‐cultured with different treatments (25 µg mL^−1^) for 3 h, respectively. Subsequently, the cultures were centrifuged at 8000 rpm for 10 min at 4°C. The residual drugs were washed away under aseptic conditions, and the bacterial cells were collected. The changes in bacterial gene expression after treatment with different drugs were measured by RNA sequencing analysis in Shanghai Personal Biotechnology Co. Ltd. Finally, the data were analyzed by using Personalbio GenesCloud (https://www.genescloud.cn) and ChiPlot (https://www.chiplot.online/).

### 4.32 Mouse Infection Model

C57BL/6J (RRID: IMSR_JAX:000664, 3–4 weeks, female) mice were purchased from Charles River Laboratories (Zhejiang) Co. Ltd. First, mouse models infected with *H. pylori* were established. C57BL/6J mice were randomly allocated into two groups (6 in the healthy control group, 24 in *H. pylori*‐infected group). The mice in the healthy group were gavaged with Brucella Broth, while the mice in the infected group were orally administered with a bacterial solution at a concentration of 3 × 10^8^ CFU mL^−1^ for three consecutive times. One week after the *H. pylori* infection, the gastric infection status of the mice was detected using standard plate counting assay, H&E staining, and qPCR.

Upon confirming the successful colonization of *H. pylori*, the infected C57BL/6J mice were randomly divided into three groups, with each group consisting of eight mice (PBS group, TBF group, and Q.T. group). Subsequently, the mice in the healthy group and PBS group were gavaged with PBS, while the TBF group was administered with 10 mg mL^−1^ of TBF, and the Q.T. group was given a combination of four drugs (303 mg kg^−1^ amoxicillin, 61 mg kg^−1^ levofloxacin, 6 mg kg^−1^ esomeprazole, and 182 mg kg^−1^ bismuth potassium citrate) via gastric gavage for five consecutive days. The mice were sacrificed 1 day after the last treatment for subsequent biochemical experiments. A portion of the gastric tissues was homogenized, diluted, and spread onto culture plates to assess the in vivo antibacterial activity. The gastric tissue specimens fixed with formaldehyde were dehydrated and embedded in paraffin for histological staining analysis. Tissue sections with a thickness of 5 µm were prepared using a microtome for H&E staining, Giemsa staining, and immunofluorescence staining.

### 4.33 Urease Assay

The urease assay was performed using the phenol red method as described previously [57]. Samples of equal mass from different treatment groups were added to the reaction reagent containing 2% urea, 0.04% phenol red, 0.04% sodium dihydrogen phosphate, and 0.1% disodium hydrogen phosphate. The reaction was carried out at room‐temperature, and the absorbance at 570 nm was measured over time.

### 4.34 qPCR

An appropriate amount of gastric tissue samples from different experimental groups were added to 1 mL of pre‐cooled RNA extraction reagent and ground thoroughly until no visible tissue clumps remained. Subsequently, the mixture was centrifuged at 12 000 rpm for 10 min to obtain the supernatant. Then, 250 µL of chloroform was added to the centrifuge tube. The tube was inverted and shaken gently for 15 s to ensure thorough mixing of the solution, and then centrifuged at 12 000 rpm for 10 min at 4°C. The resulting supernatant was mixed completely with isopropanol, placed at −20°C for 15 min, and then centrifuged again at 12 000 rpm for 10 min at 4°C. As a result, white precipitated RNA was obtained. After eliminating the DNA component, reverse transcriptase was added to synthesize cDNA. Subsequently, specific primers, which are listed in Table S2, were added for performing qPCR. For bacterial qPCR analysis, log‑phase *H. pylori* (1 × 10^8^ CFU mL^−1^) were co‑incubated with TBF (25 µg mL^−1^) for 6 h before RNA extraction, followed by qPCR using the same procedure described above.

### 4.35 Mucosa Permeation Studies

The transport of TBF across the mucosa was investigated using the Transwell system assay. In brief, 250 mg of mucosa harvested from freshly slaughtered pigs was evenly spread on a polycarbonate membrane with a pore size of 3 µm. Meanwhile, 1 mL of *H. pylori* suspension was placed in the receptor chamber. Subsequently, 2 mL of fluorescently labeled TBF (TBF‐RhB) was carefully added to the surface of the mucosa. The entire plate was then incubated in a shaker at 37°C with a rotation speed of 100 rpm. Samples of 200 µL were taken from the receptor chamber over time, and the absorbance at 600 nm was measured. Eventually, the TBF‐RhB in the receptor chamber was collected for fluorometric analysis. The apparent permeability coefficient (P_app_) was calculated using the equation provided below:

$$\;P_{\mathrm{app}}=\frac{\mathrm{dQ}}{\mathrm{dt}}\;\times \frac{1}{A}\times C_{0}\times 100\%$$

where dQ/dt is the flux of fluorescently labeled TBF from the donor side to the acceptor side, C_0_ is the initial concentration of TBF in the donor compartment, and A is the membrane area (cm^2^).

### 4.36 In Vivo Imaging Experiments

The mouse model infected with *H. pylori* was first established. After fasting the mice for 12 h, 300 µL of fluorescently labeled nanodrugs (TB‐RhB, TBF‐RhB) were administered to the mice by gavage. Subsequently, the fluorescence distribution in the mice was observed at different time points (4600SF, Tanon, China).

### 4.37 Bi^3+^ Release Assay

5 mL of TBF suspension (10 mg mL^−1^) was placed into a dialysis bag with a molecular weight cutoff of 3.5 kDa, which was immersed in 60 mL of PBS (pH 7.4) and SGF pH 4.5), respectively. The systems were incubated at 37°C with shaking at 180 rpm. At predetermined time points (0, 1, 2, 3, 4, 5, 6 and 12 h), 1 mL of dialysate was collected, and an equal volume of fresh blank medium was replenished. The Bi^3+^ concentration in the dialysate at each time point was quantified using inductively coupled ICP‑MS.

### 4.38 Bactericidal Effect of TBF Under Different pH Conditions

TBF was pre‑incubated in PBS and SGF for 3 h at a final concentration of 500 µg mL^−1^. Log‑phase *H. pylori* were then co‑cultured with pre‑treated TBF, and colony numbers were quantified by the plate‑counting method. Meanwhile, *H. pylori* were resuspended in Brucella Broth (containing 10% FCS) at different concentrations and treated with TBF at serial concentrations of 0, 0.39, 0.78, 1.56, 3.12, 6.25, 12.5, 25 and 50 µg mL^−1^. After incubation for 72 h in a tri‑gas incubator, the absorbance at 600 nm was measured, and the bacterial viability was calculated using equation provided below:

$\mathrm{Bacterial}\;\mathrm{viability}(\%)\;=\frac{A_{600s}-A_{600b}}{A_{600c}-A_{600b}}\;\times 100\%$

Where A_600b_ is the absorbance at 600 nm of the blank culture medium without bacteria, A_600s_ is the absorbance of the drug‑treated group at 600 nm, A_600c_ is the absorbance of the control group at 600 nm.

### 4.39 Scavenging In Vitro ROS

The in vitro reactive oxygen species (ROS) scavenging abilities of different nanomaterials were measured using the DCFH‐DA method as described previously [58]. First, 50 µm of the ROS probe DCFH‐DA was allowed to react with 5 mm of H_2_O_2_ for 5 min. Subsequently, 10 µL of various nanodrugs was added, and the mixture was incubated at 37°C for 40 min. Following that, the fluorescence peak at 525 nm was measured using a fluorescence photometer (SpectraMax i3x, Molecular Devices, United States).

### 4.40 Western Blot Analysis

An appropriate amount of gastric tissue was collected from different experimental groups. These samples were then homogenized and ground to extract the tissue proteins, which were subsequently quantified using the BCA method. A total of 30 µg protein per well was loaded onto either an 8% or a 10% SDS‐PAGE gel, separated by electrophoresis, and transferred onto a polyvinylidene fluoride (PVDF) membrane. The PVDF membrane was then incubated overnight with the following primary antibodies: JAK2 (1:1000, RRID: AB_3675337, Wanleibio), pSTAT3 (1:1000, RRID: AB_1658549, Abcam), IL‐6 (1:1000, RRID: AB_2936896, Abmart), ZO‐1 (1:1000, RRID: AB_2936803, Abmart), E‐Cadherin (1:1000, RRID: AB_2936787, Abmart), and β‐actin (1:5000, RRID:AB_2223210, Abcam). Subsequently, the membrane was incubated with an appropriate secondary antibody. Finally, the protein expression levels were evaluated by analyzing the grayscale intensity of the corresponding protein bands on the membrane (4600SF, Tanon, China).

### 4.41 The In Vitro Impact of TBF on the Viability of Probiotic Bacteria

The probiotic bacteria stored at −80°C were thawed in a 37°C‐water bath. Subsequently, a small volume of the bacterial suspension was transferred into the MRS medium. After undergoing two rounds of activation within an anaerobic incubator, various nanodrugs were added to the bacterial suspension. The OD_600_ values of the suspension were measured both before and 18 h after the incubation process. The bacterial viability was calculated using the equation provided below:

$\mathrm{Bacterial}\;\mathrm{viability}(\%)\;=\frac{OD_{600a}-OD_{600c}}{OD_{600b}-OD_{600c}}\;\times 100\%$

Where OD_600a_ is the absorbance of the culture system at 600 nm after co‐culturing with the drugs for 18 h, OD_600b_ is the absorbance value of the culture system at 600 nm before co‐culturing with the drug for 18 h, and OD_600c_ is the absorbance value of the blank control group without adding bacteria at 600 nm.

### 4.42 Gut Microbiota Analysis

After different treatments, the feces of the mice were carefully collected. Subsequently, both the abundance and composition of the microbial community, along with the metabolic status of SCFAs in the mouse feces, were determined through 16S rRNA sequencing and targeted metabolomics analysis conducted by APExBIO Technology LLC.

### 4.43 Statistical Analysis

Experimental data were statistically analyzed, with results expressed as mean ± standard deviation (SD). Each statistical analysis was conducted with a minimum sample size of 3. For comparisons between two groups, statistical differences were assessed using two‐sided Student's *t*‐tests. When analyzing differences across three or more groups, one‐way analysis of variance (ANOVA) followed by post‐hoc Tukey's test was employed. A *p*‐value < 0.05 was defined as statistically significant in all analyses. Graphing and statistical analyses were performed using OriginPro 2025b (Learning Edition).

## Author Contributions

**Tianye Fang**: conceptualization, methodology, data curation, validation, investigation, writing – original draft, writing – review and editing. **Jinzhe Tong**: methodology, data curation, validation, investigation. **Feng Feng**: methodology, investigation, validation, writing – review and editing. **Cong Liu**: conceptualization, methodology. **Chang Shu**: conceptualization, methodology. **Jiaying Zhu**: investigation. **Shibo Zhang**: methodology, investigation. **Shuyue Deng**: writing – review and editing. **Wanchao Zuo**: methodology, investigation. **Yuhan Song**: investigation. **Jun Yang**: resources. **Yanmin Ju**: resources, supervision, funding acquisition, writing – review and editing. **Yingying Xing**: resources, supervision. **Jianjun Dai**: resources, supervision, funding acquisition.

## Funding

This work was financially supported by the National Natural Science Foundation of China (No. 32172855 and No. 22574173) and the Project Program of State Key Laboratory of Natural Medicines (China Pharmaceutical University) (No. SKLNMZZ2024JS46 and No. SKLNMZZ202510).

## Conflicts of Interest

The authors declare no conflicts of interest.

## Supporting information




**Supporting file**: advs76621‐sup‐0001‐SuppMat.docx

## Data Availability

The data that support the findings of this study are available from the corresponding author upon reasonable request.
