# Supplementary material for: Disrupting Helicobacter pylori Iron Homeostasis With Bismuth Nanodrug‐Mediated Nutritional Trap for Targeted Gastric Infection Therapy
Source: Adv Sci (Weinh). 2026 Jul 20:e76621. Online ahead of print. doi: 10.1002/advs.76621 (PMC13383705; doi:10.1002/advs.76621)
Supplement: Supplementary file 1 — Supporting file: advs76621‐sup‐0001‐SuppMat.docx [file ADVS-9999-e76621-s001.docx]

Supporting Information

Disrupting *Helicobacter pylori* Iron Homeostasis with Bismuth Nanodrug-Mediated Nutritional Trap for Targeted Gastric Infection Therapy

Tianye Fang, Jinzhe Tong, Feng Feng, Cong Liu, Chang Shu, Jiaying Zhu, Shibo Zhang, Shuyue Deng, Wanchao Zuo, Yuhan Song, Jun Yang, Yanmin Ju*, Yingying Xing*, and Jianjun Dai*

*Email: [juyanmin@cpu.edu.cn](mailto:juyanmin@cpu.edu.cn); [jjdai@cpu.edu.cn](mailto:jjdai@cpu.edu.cn); [xingyy@cpu.edu.cn](mailto:xingyy@cpu.edu.cn)

**This supplement includes:**

[Supplementary Figures 2](#_Toc232107964)

[Supplementary Tables 16](#_Toc232107965)

**Supplementary Figures**


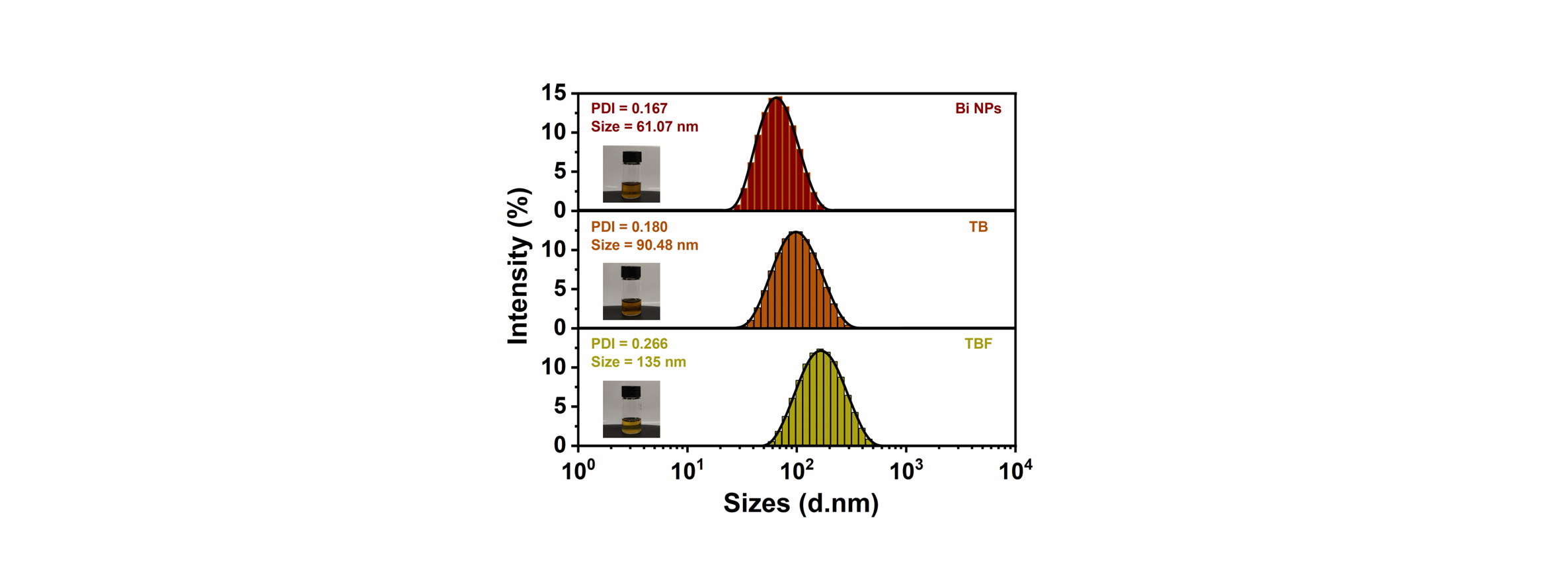


**Figure S1.** Particle diameter and typical images of Bi NPs, TB and TBF.


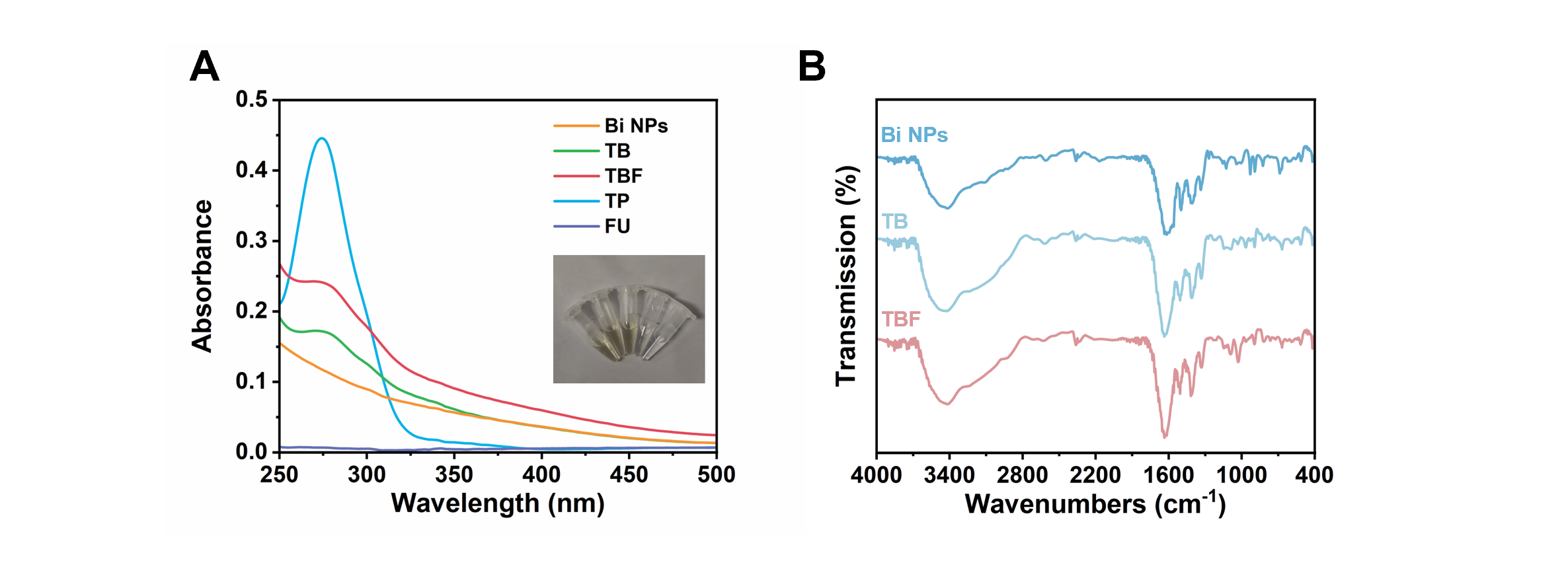


**Figure S2.** The spectral measurement results of each component of the nanodrug. (A) UV-vis spectrum analysis of Bi NPs, TB, TBF, TP and FU. (B) FTIR spectrum analysis of Bi NPs, TB and TBF.


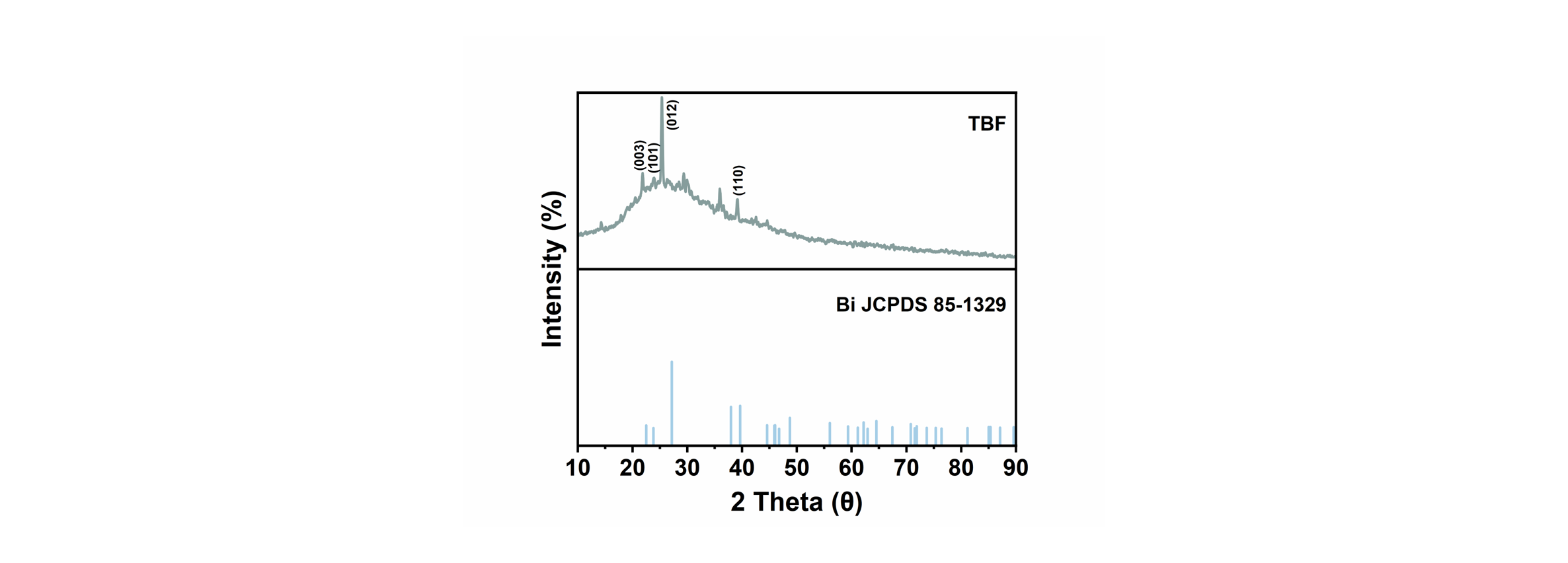


**Figure S3.** XRD patterns of TBF.


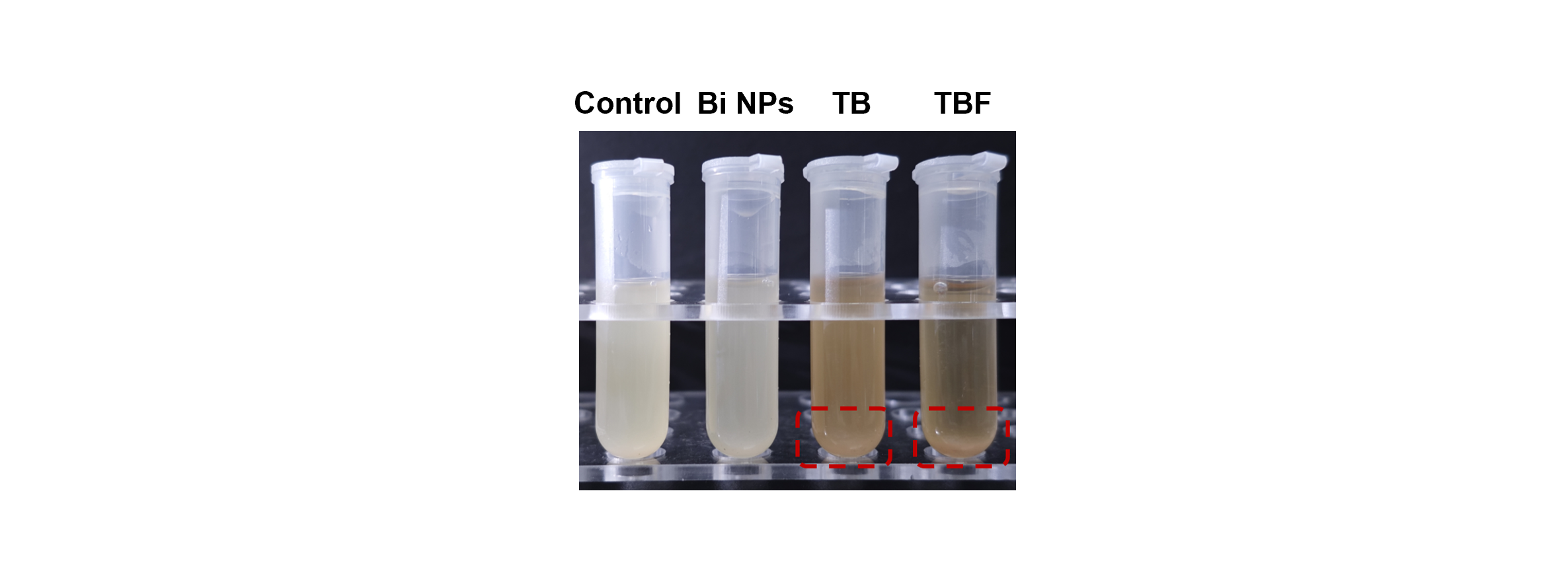


**Figure S4.** Typical photographs of the whole culture system after co-culture of different nanodrugs with *H. pylori*.


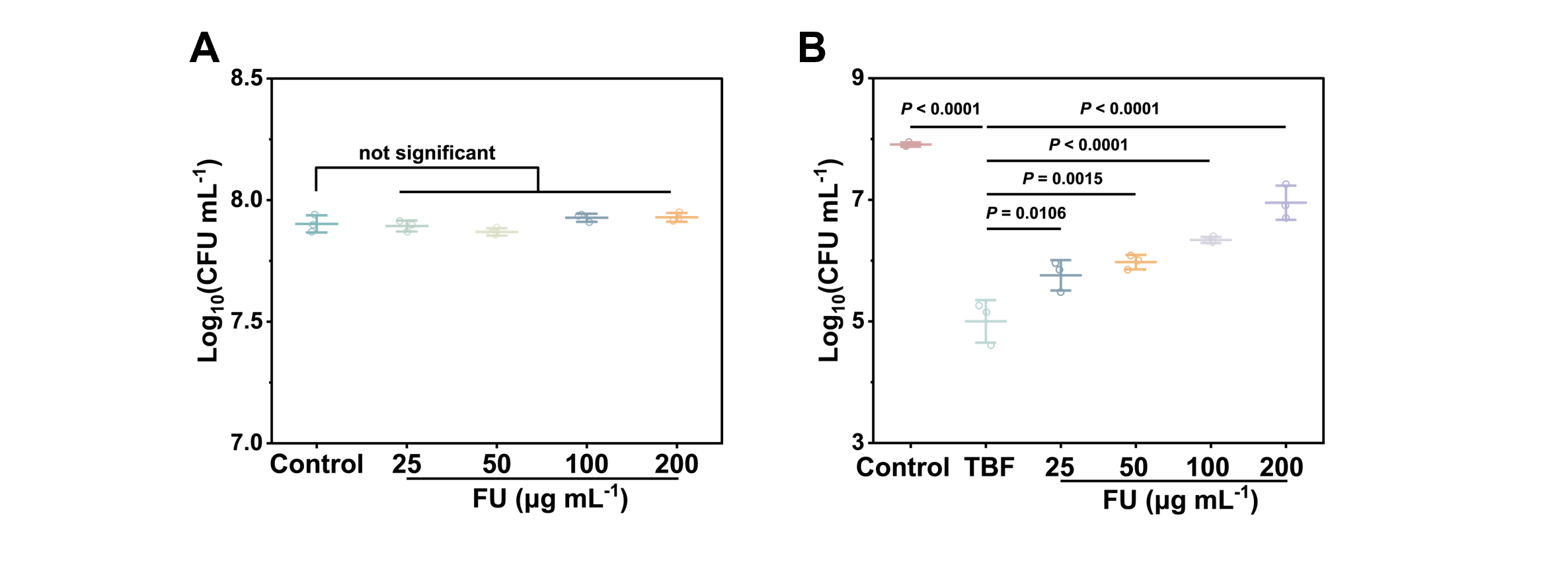


**Figure S5.** Validation of antibacterial efficacy via fucoidan-mediated BabA-specific targeting. (A) Plate counting results of *H. pylori* co-cultured with fucoidan. (B) Plate counting results of *H. pylori* pre-treated with fucoidan followed by TBF. Data are presented as mean ± SD. n = 3, biological replicates. One-way ANOVA with Tukey’s post hoc testing was used for statistical analysis.


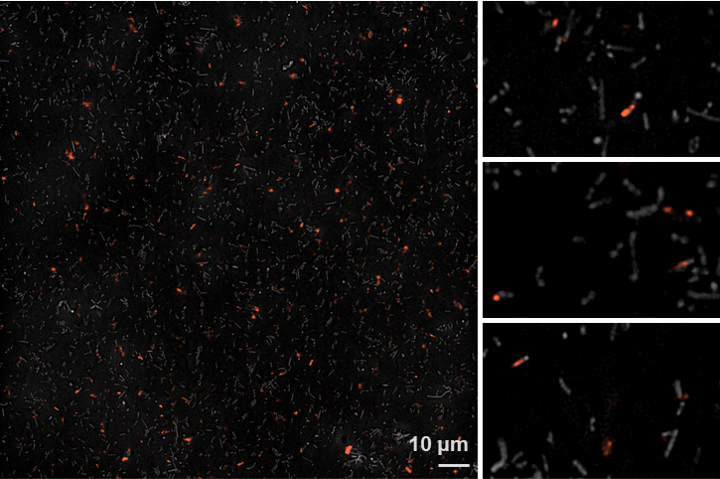


**Figure S6.** ODT images of *H. pylori* merged with TBF fluorescence.


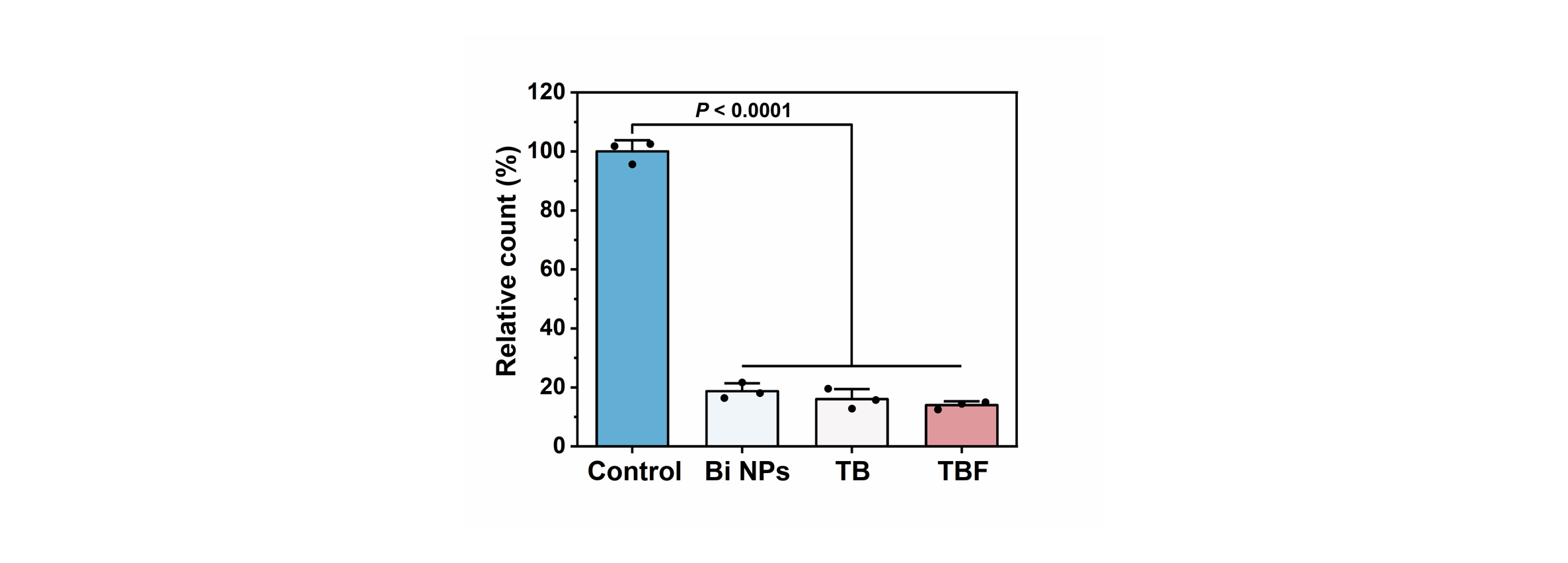


**Figure S7.** Bacterial ATPase activity after different nanodrug treatments. Data are presented as mean ± SD. n = 3, biological replicates. One-way ANOVA with Tukey’s post hoc testing was used for statistical analysis.


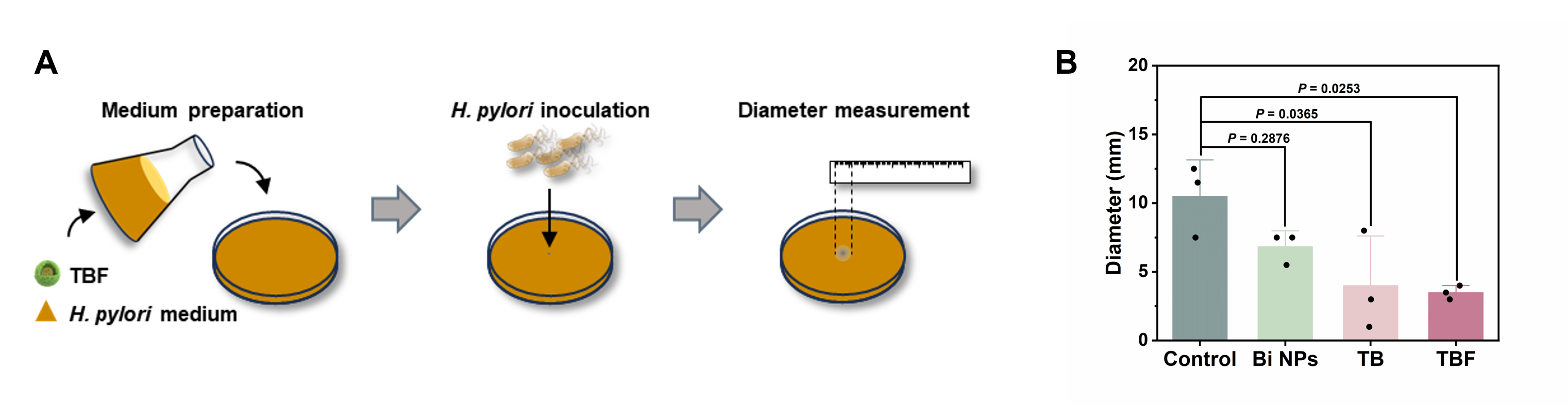


**Figure S8.** Motility inhibition of TBF on *H. pylori*. (A) Schematic diagram of bacterial mobility test. (B) Statistics of plaque diameter size after treatment with different nanodrug groups. Data are presented as mean ± SD. n = 3, biological replicates. One-way ANOVA with Tukey’s post hoc testing was used for statistical analysis.


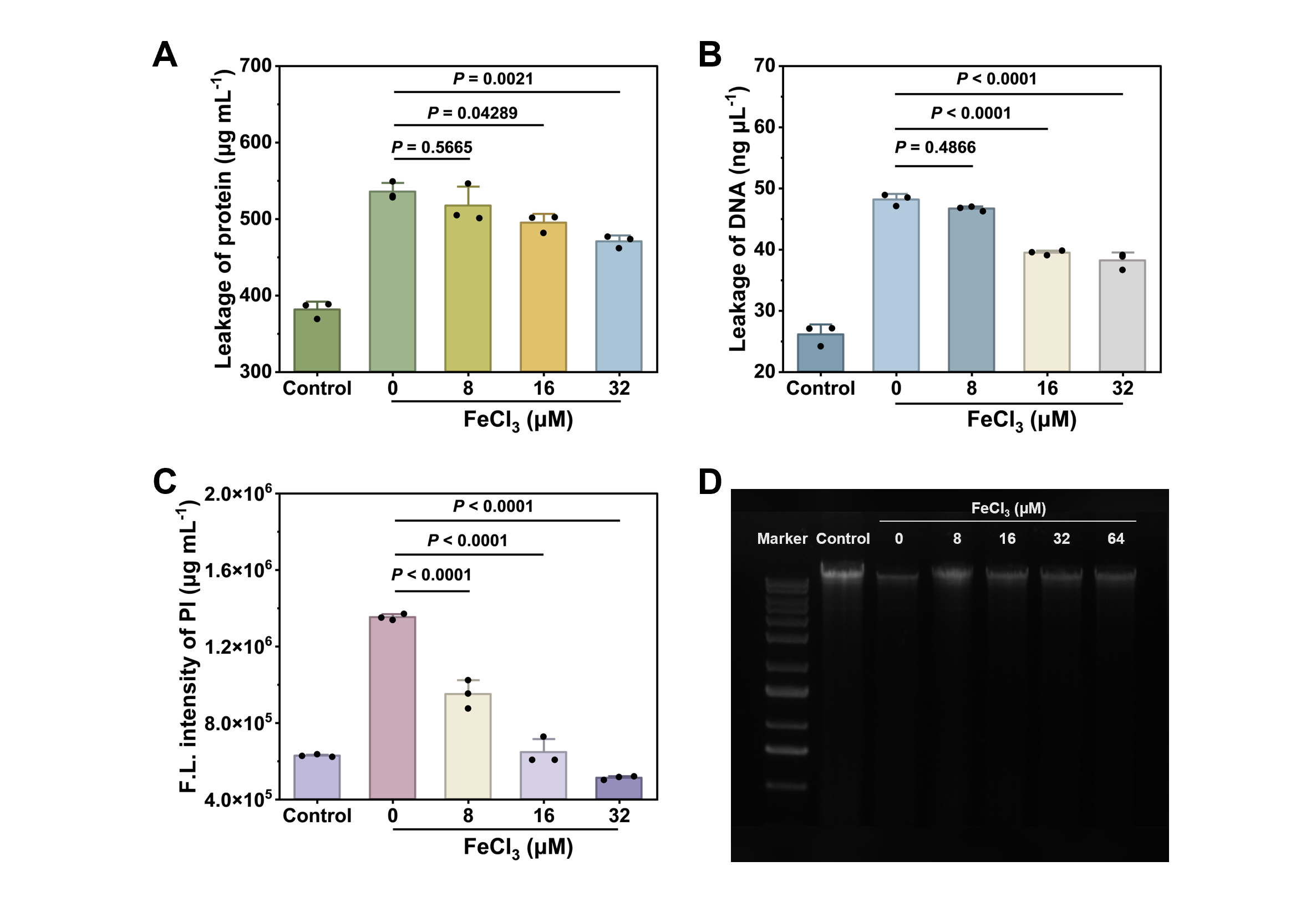


**Figure S9.** Validation of iron homeostasis disruption as the dominant causal mechanism for TBF-mediated bactericidal effects on *H. pylori* via iron supplementation rescue assays. (A) Protein leakage and (B) DNA release of *H. pylori* after TBF treatment with supplementary FeCl_3_. (C) PI fluorescence intensity following TBF intervention combined with FeCl_3_ addition. Data are presented as mean ± SD. n = 3, biological replicates. One-way ANOVA with Tukey’s post hoc testing was used for statistical analysis. (D) Agarose gel electrophoresis images of *H. pylori* genomic DNA after TBF treatment with the addition of FeCl_3_.


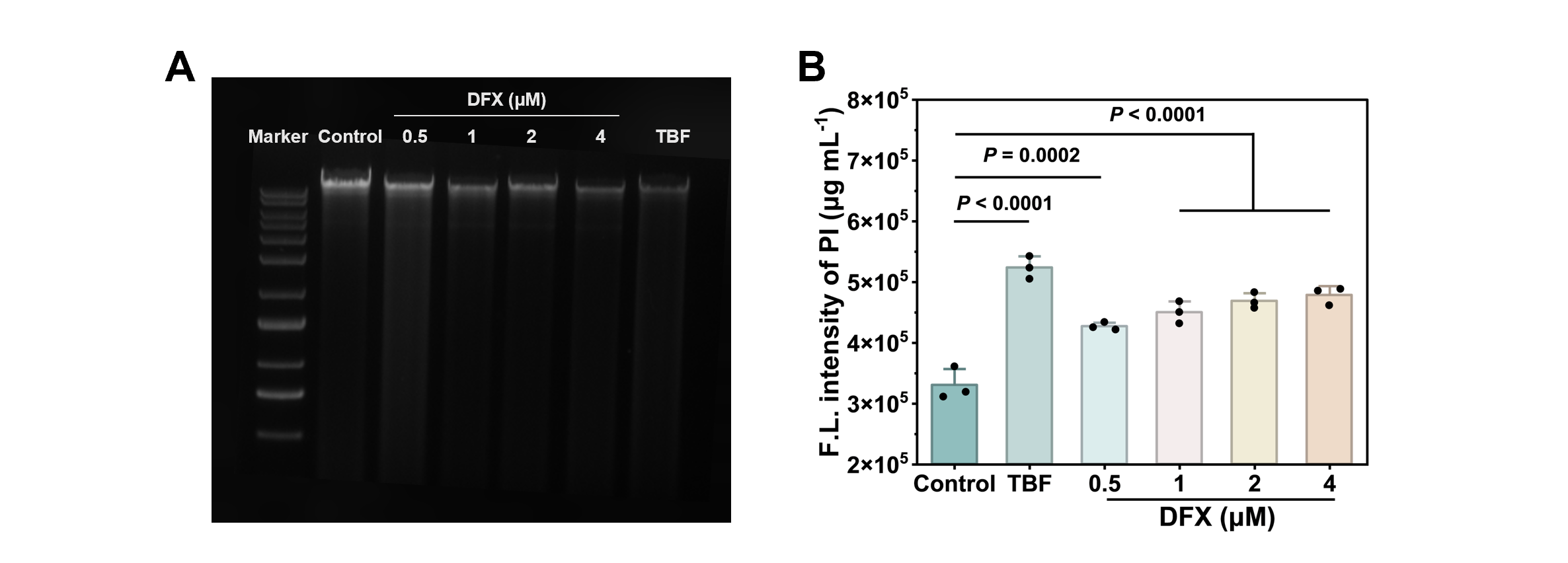


**Figure S10.** Validation of iron homeostasis disruption as the dominant causal mechanism for TBF-mediated bactericidal effects on *H. pylori* via positive drug control. (A) Agarose gel electrophoresis images of *H. pylori* genomic DNA after DFX treatment. (B) PI fluorescence intensity detected after DFX treatment. Data are presented as mean ± SD. n = 3, biological replicates. One-way ANOVA with Tukey’s post hoc testing was used for statistical analysis.


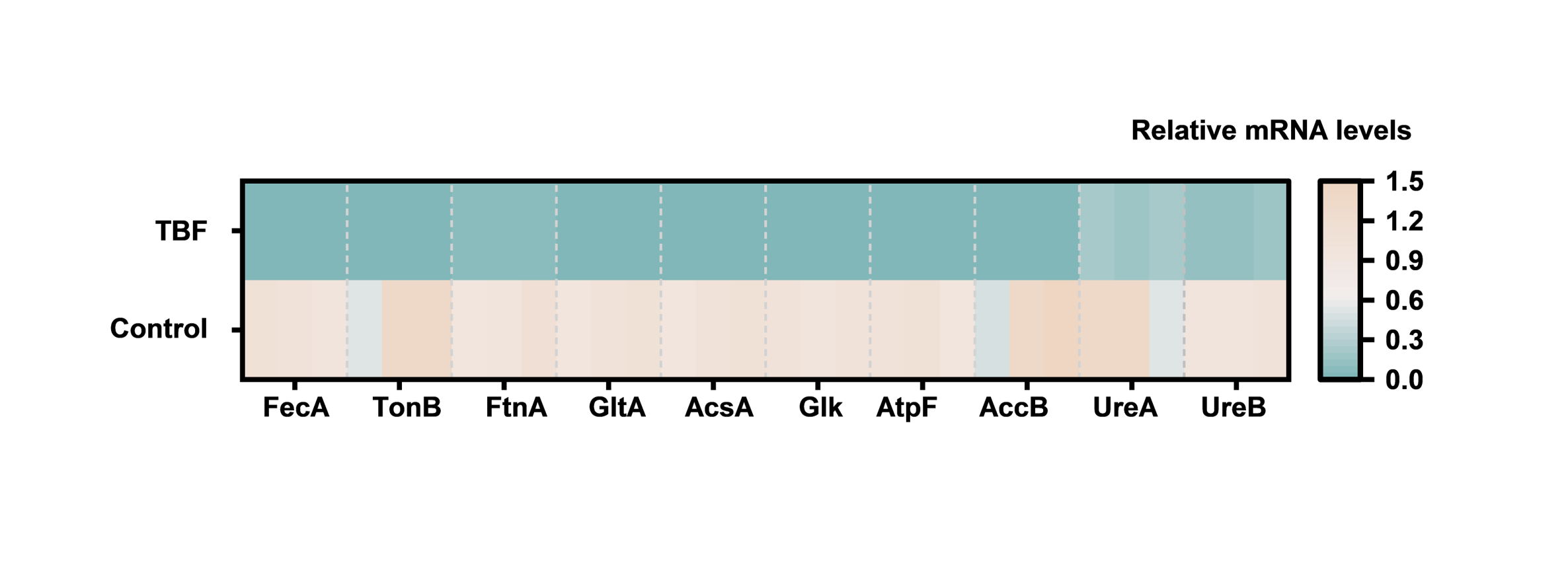


**Figure S11.** qPCR results of iron homeostasis‑related genes in *H. pylori* after TBF treatment. n = 3, biological replicates.


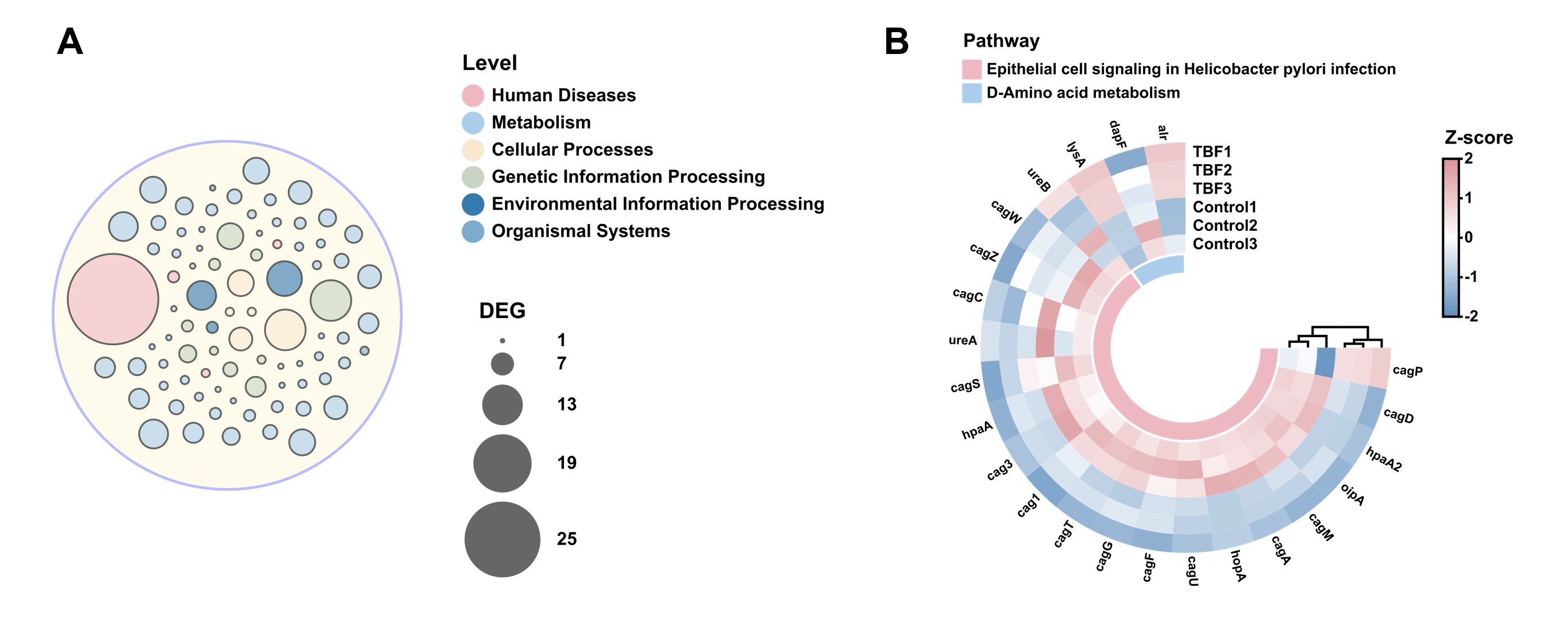


**Figure S12.** KEGG pathways associated with DEGs of *H. pylori* after TBF treatment. (A) KEGG annotation analysis of DEGs in *H. pylori* treated with TBF. (B) Heatmap of DEGs involved in epithelial cell signaling in *H. pylori* infection and D-Amino acid metabolism.


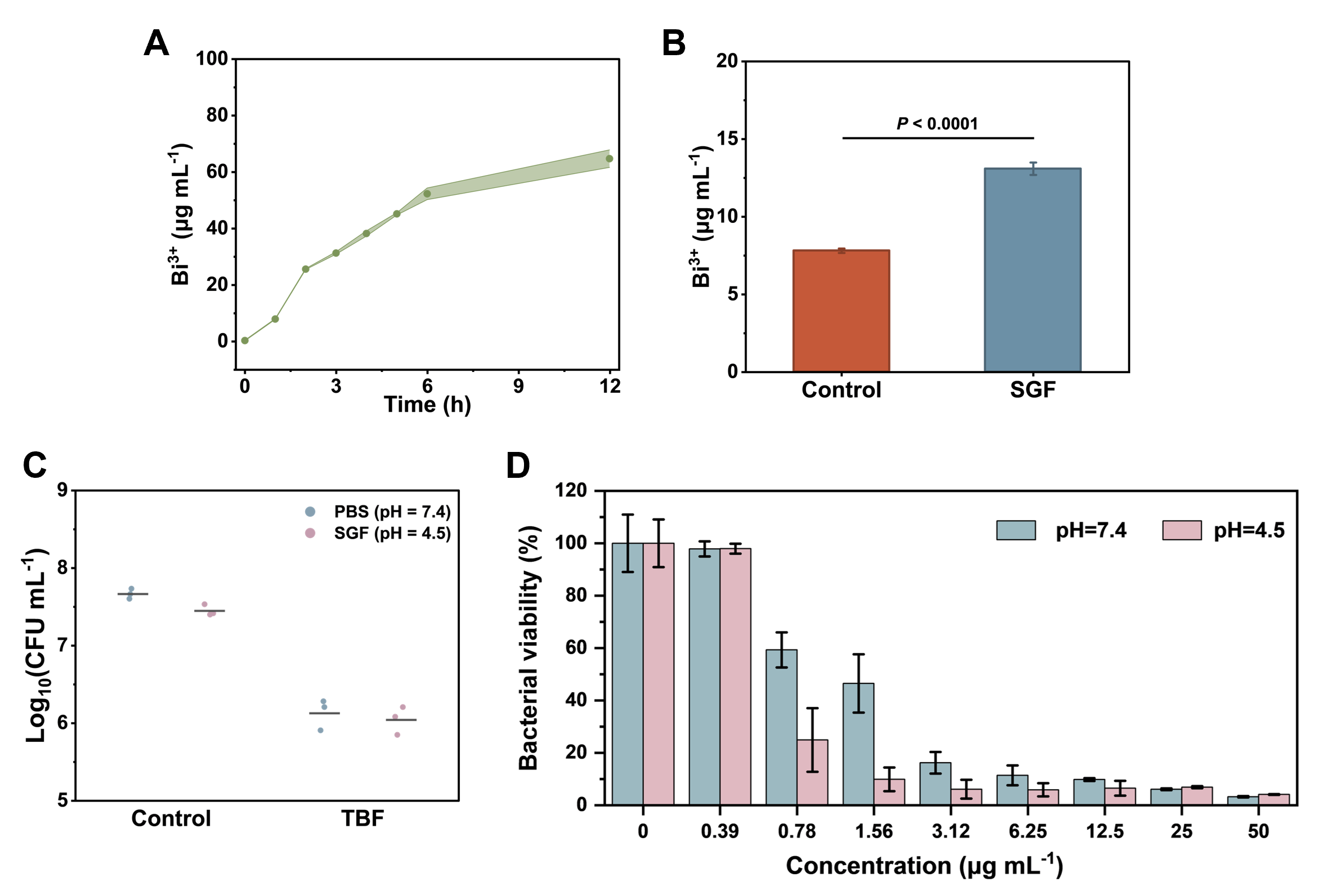


**Figure S13.** Characterization of Bi^3+^ release and pH‑dependent antibacterial activity of TBF. (A) Time‑dependent release profile of Bi^3+^ from TBF under simulated gastric peristalsis. (B) Comparison of Bi^3+^ release amounts from TBF after 1 h dialysis in PBS (Control) and SGF. (C) Plate counting results of *H. pylori* after co-culture with TBF pretreated in PBS (pH = 7.4) and SGF (pH = 4.5) for 3 h. (B) Viability of *H. pylori* treated with TBF at different concentrations under neutral and acidic conditions. Data are presented as mean ± SD. n = 3, biological replicates. One-way ANOVA with Tukey’s post hoc testing (A, C, D) and two-sided Student’s t-tests (B) was used for statistical analysis.


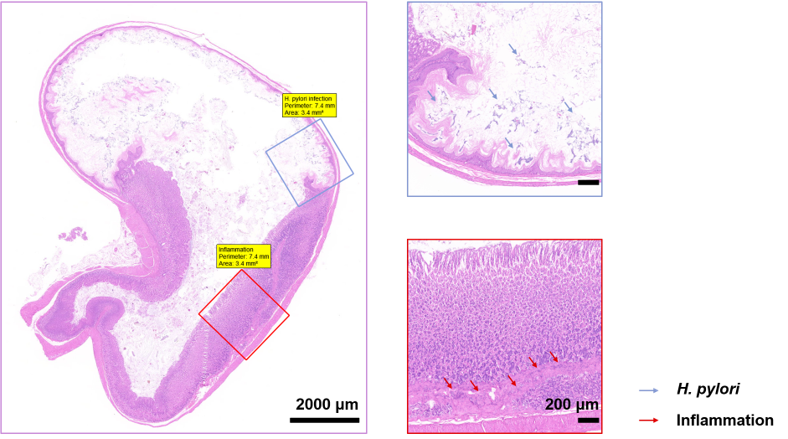


**Figure S14.** Representative panoramic scanning images of gastric tissue sections H&E staining from *H. pylori* infected mice. Blue box: representative areas of *H. pylori* infection. Blue arrow: *H. pylori*. Red box: representative areas of inflammation. Red arrow: inflammatory infiltrate.


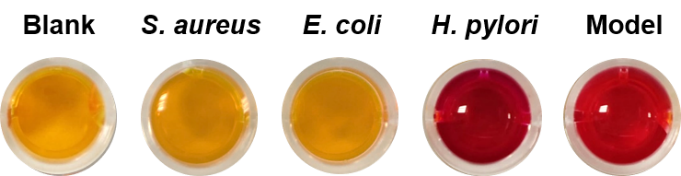


**Figure S15.** Urease assay in the mouse model of *H. pylori* infection.


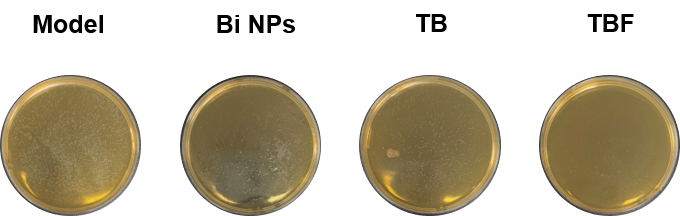


**Figure S16.** Representative photographs of plates coated with mouse gastric tissue homogenates treated with Bi NPs, TB, TBF.


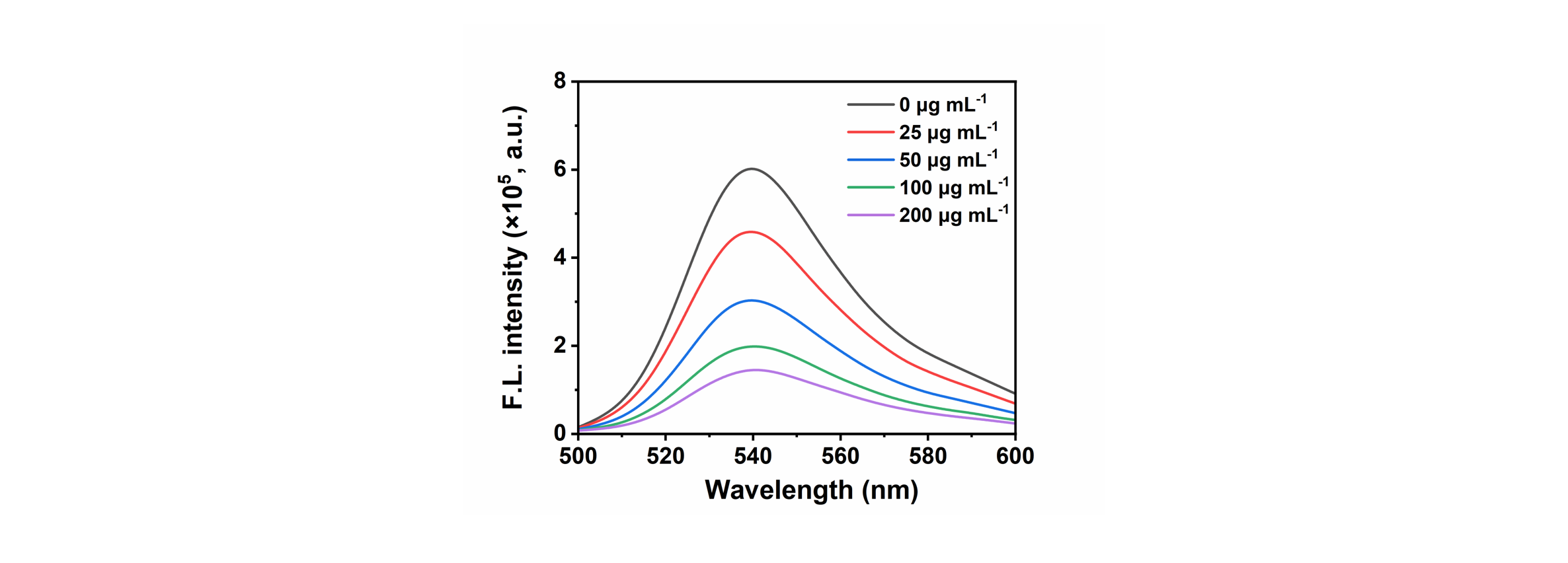


**Figure S17.** The fluorescence intensity of the system after treatment with different concentrations of TBF detected using the DCFH-DA probe.


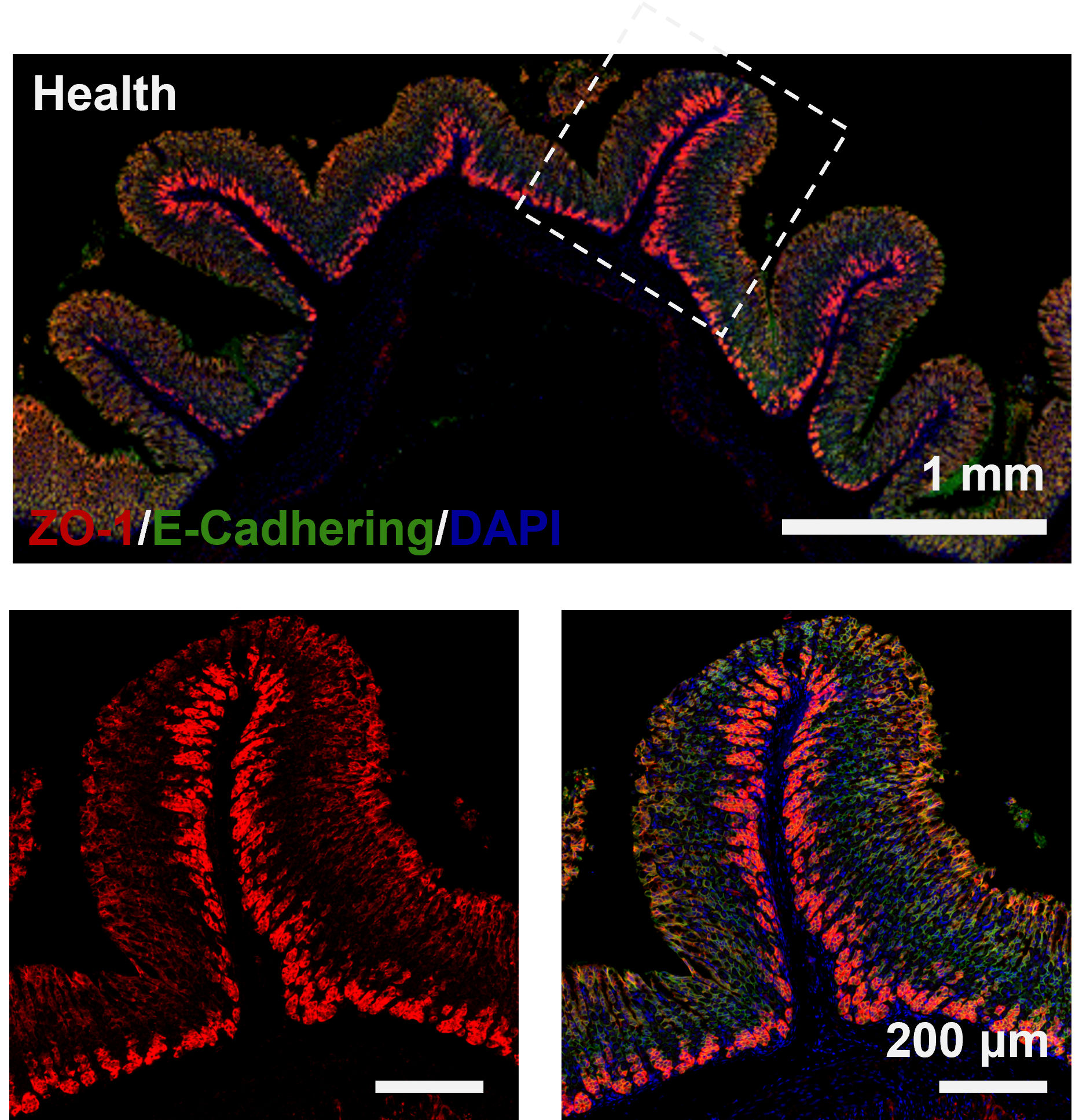


**Figure S18.** Representative images of immunofluorescence of health group. Red for ZO-1, Green for E-Cadherin, and Blue for DAPI.


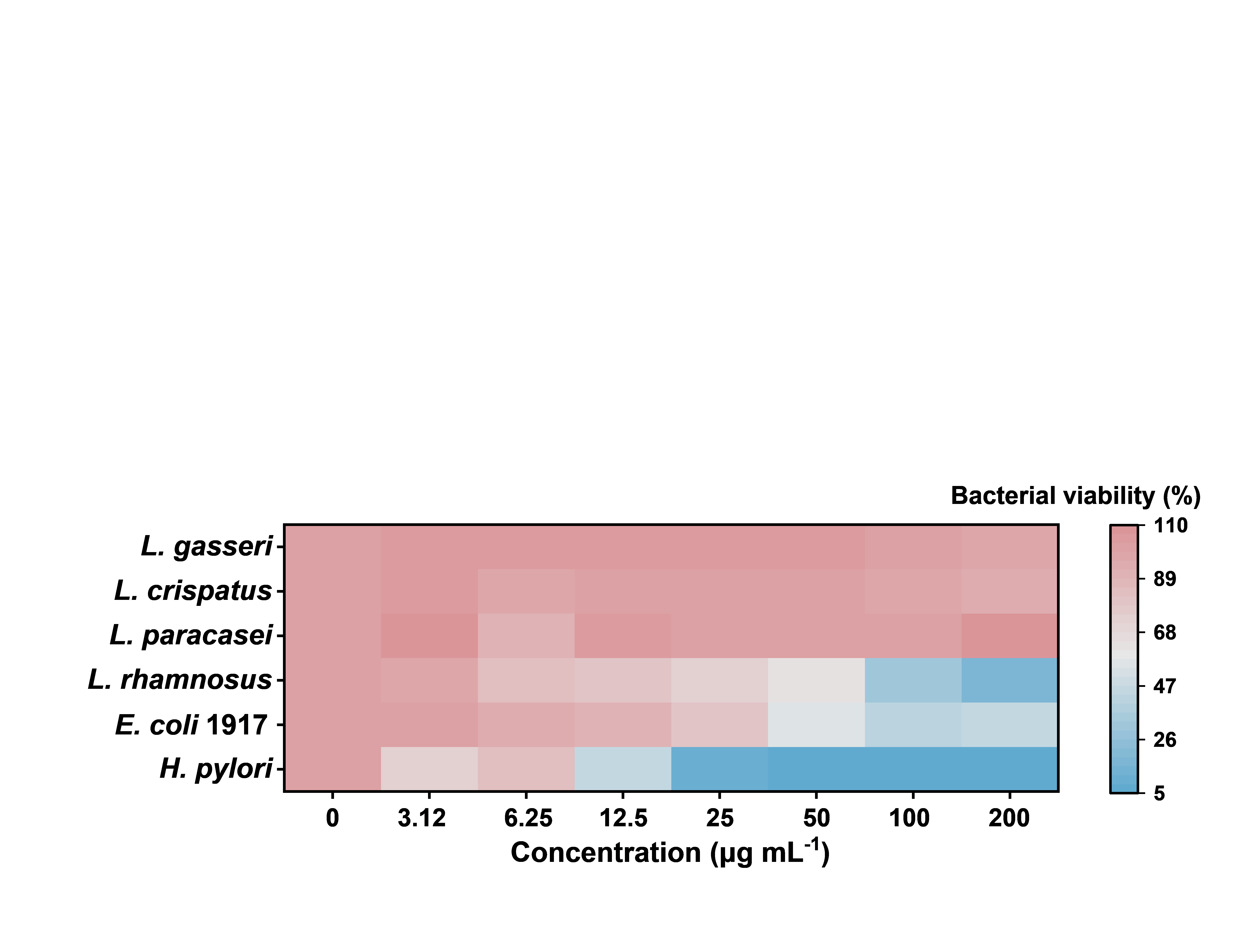


**Figure S19.** Survival rate of *L. gasseri*, *L. crispatus*, *L. paracasei*, *L. rhamnosus*, *E. coli* 1917 and *H. pylori* after treatment with different concentrations of TBF.


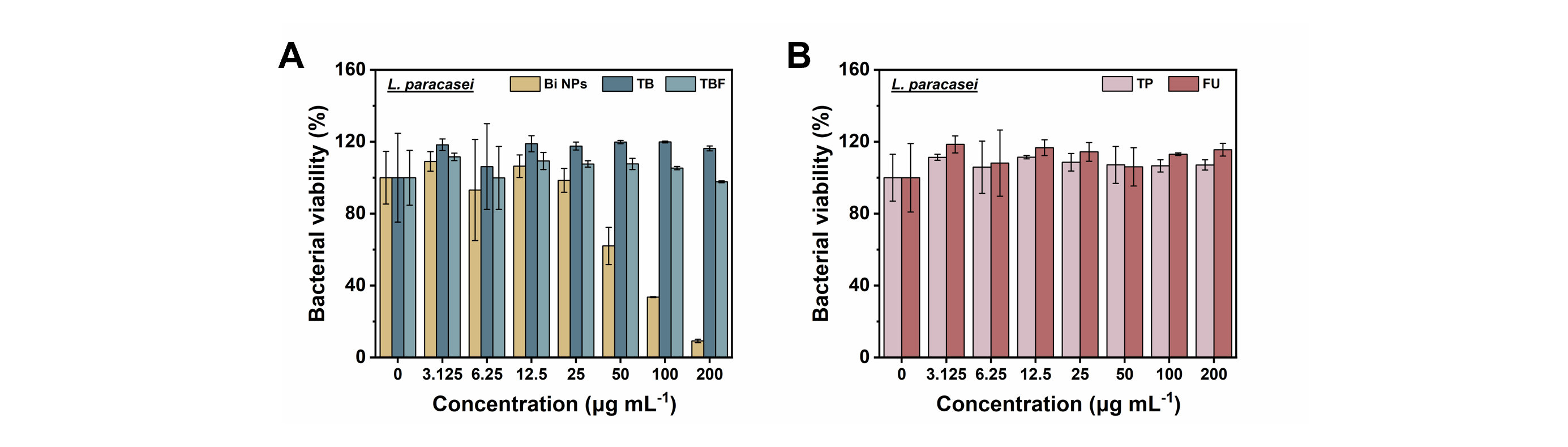


**Figure S20.** Bacterial viability of *L. paracasei* treated with Bi NPs, TB, TBF, TP and FU respectively. Data are presented as mean ± SD. n = 3, biological replicates.


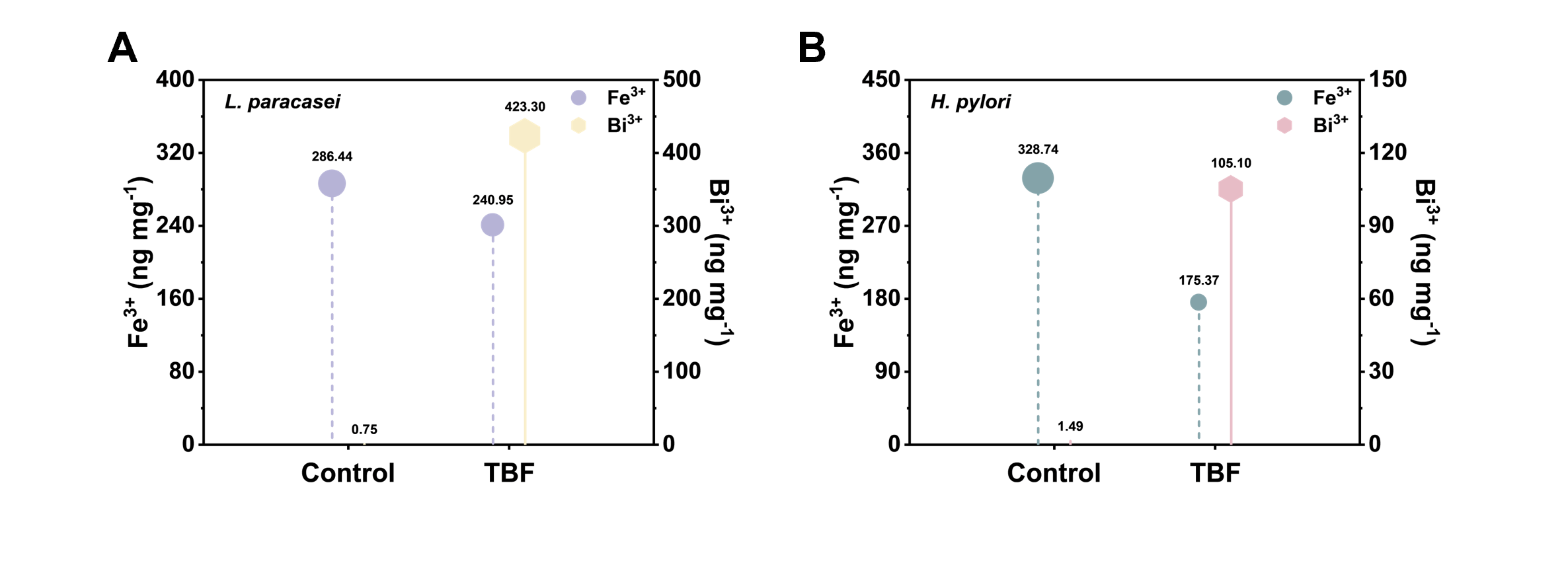


**Figure S21.** Effects of changes in intracellular ion concentrations on bacterial activity after drug administration. Content of Fe^3+^ and Bi^3+^ in (A) *L. paracasei* and (B) *H. pylori* after TBF treatment.


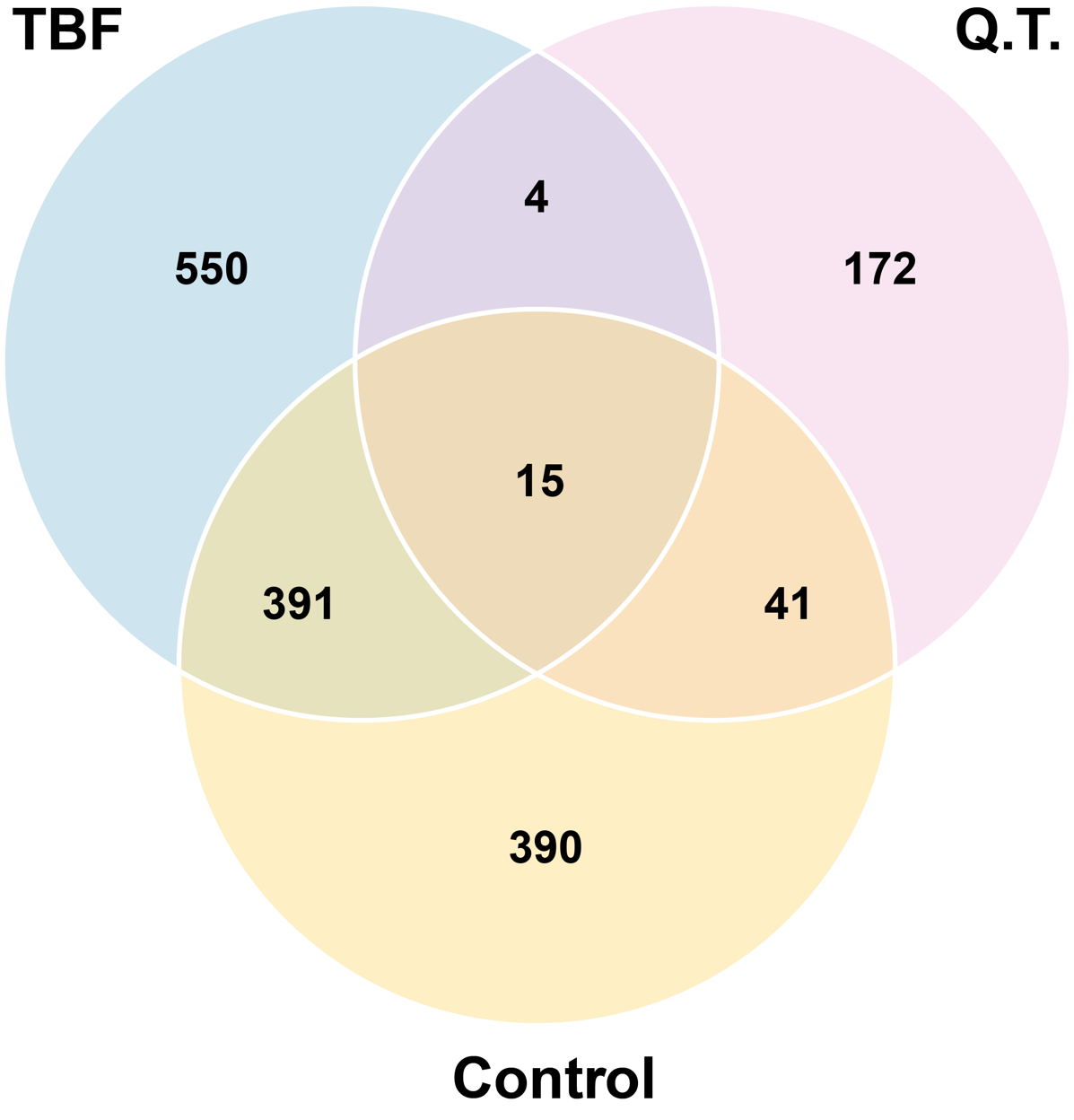


**Figure S22.** Venn diagram displaying the overlap of the amplicon sequence variants (ASV) identified in the gut microbiota between different groups.


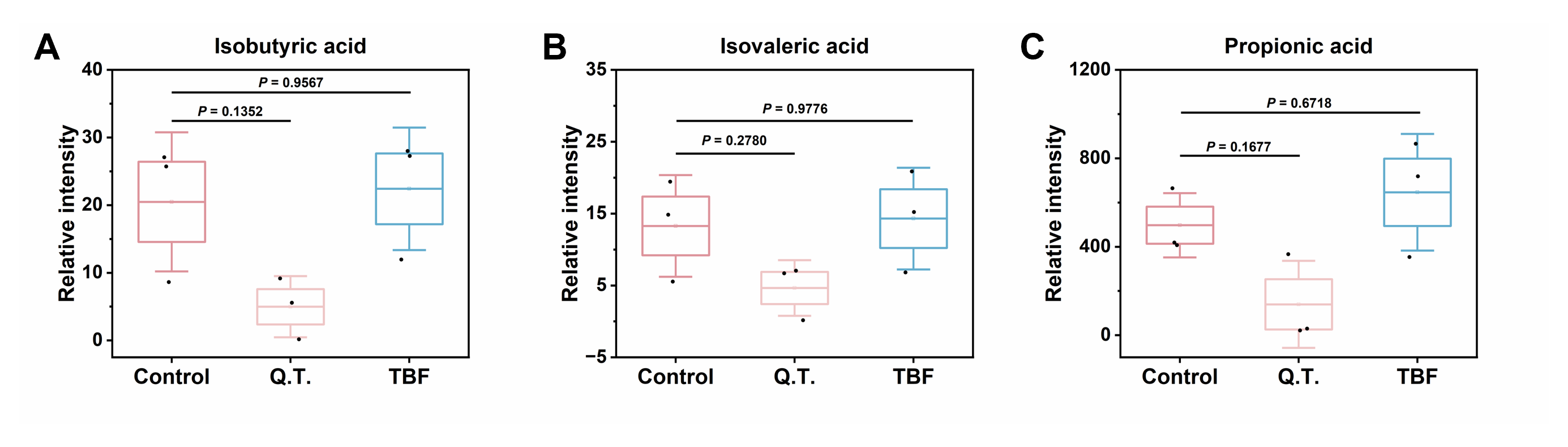


**Figure S23.** The content of three different short-chain fatty acids. Relative intensities of (A) isobutyric acid, (B) isovaleric acid and (C) propionic acid measured by targeted metabolomics. Data are presented as mean ± SD. n = 3, biological replicates. One-way ANOVA with Tukey’s post hoc testing was used for statistical analysis.


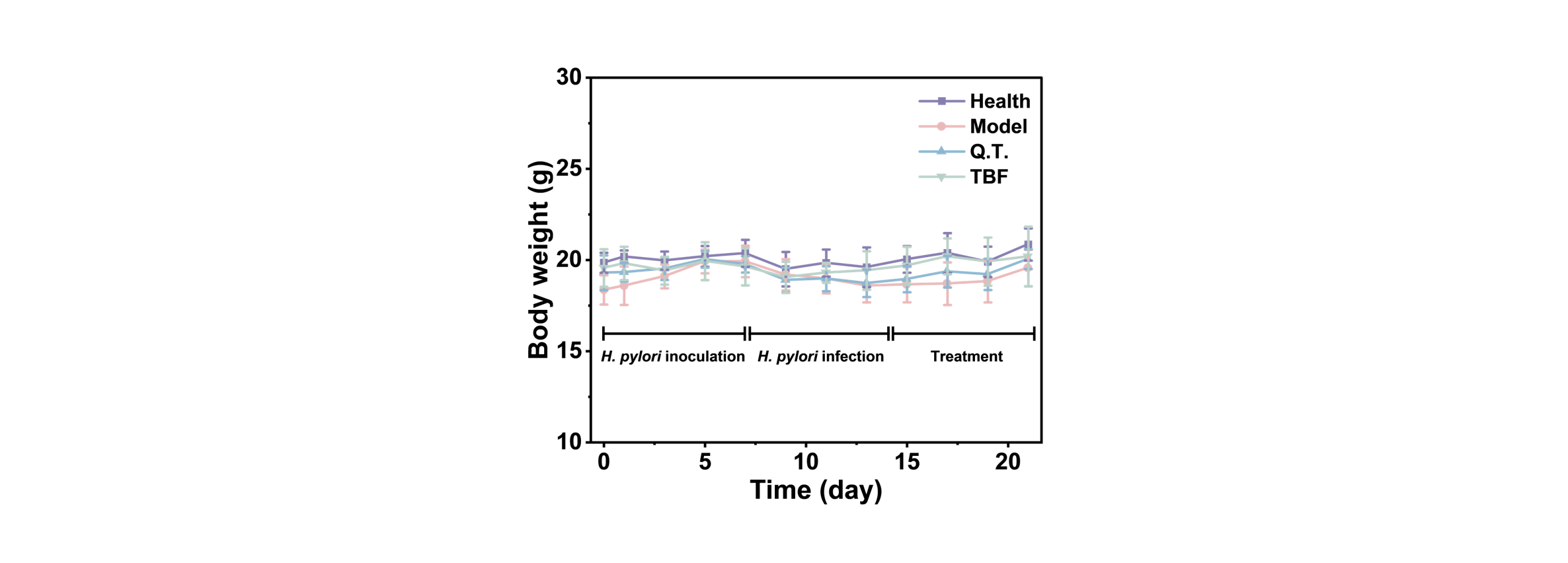


**Figure S24.** Body weight of mice during the experimental treatment period. Data are presented as mean ± SD. n = 6 in the health group, n = 8 in other groups.


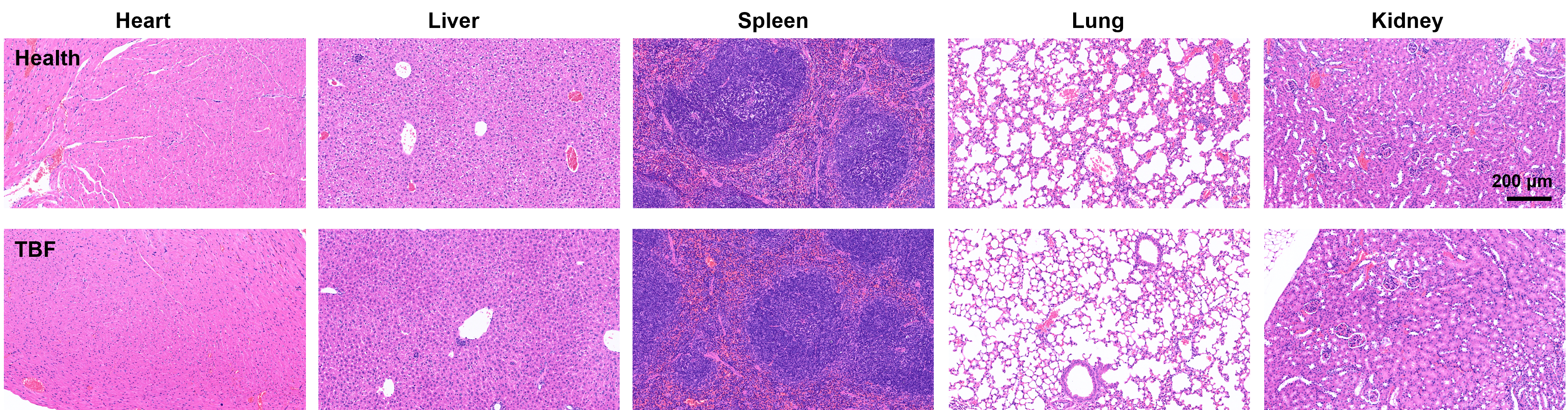


**Figure S25.** Representative images of H&E staining of mouse heart, liver, spleen, lung and kidney after TBF treatment.

**Supplementary Tables**

**Table S1.** **MIC of clinically isolated *H. pylori* strains.**

|  | NSH57  [μg mL^–1^] | SS1  [μg mL^–1^] | 4720a  [μg mL^–1^] | Resistance  [μg mL^–1^] |
| --- | --- | --- | --- | --- |
| Levofloxacin | 0.5 | 1 | 0.5 | >1 |
| Amoxicillin | 0.25 | 2 | 4 | >0.125 |
| Tetracycline | 4 | 0.5 | 0.5 | >1 |
| Clarithromycin | >8 | >64 | >64 | >0.5 |
| Metronidazole | >128 | >128 | >128 | >8 |

**Table S2. Primers used for qPCR.**

|  | Forward primer | Reverse primer |
| --- | --- | --- |
| 16s DNA | TTTGTTAGAGAAGATAATGACGGTATCTAAC | CATAGGATTTCACACCTGACTGACTATC |
| Cag A | CCCCATGTCCAACCAGATATAG | GCGACTCCCTCAACATCTAAC |
| IL-6 | AAACCGCTATGAAGTTCCTCTC | GTGGTATCCTCTGTGAAGTCTC |
| IL-17 | CCGCAATGAAGACCCTGATAG | CTTC AGGACCAGGATCTCTTG |
| INF-γ | TCTTCCTCATGGCTGTTTCTG | CACCATCCTTTTGCCAGTTC |
| UreB | GTTTGAGGGCGGATCCTTGA | GGCCGGTGAACACAACATTC |
| UreA | CATGGATCATGCTTGCCACG | GCTGGTAAAAAGACTGCGGC |
| AccB | AAAAAGGGCAAATCGTGGGC | GCGTCTCCCACTTCAACAGA |
| AtpF | GCCCTTTGTGTGCTACTGGA | ACCACAAAATCCCCGCAAAT |
| Glk | ATTTTAGGGCCAGGAACCGG | CAAAAACCTTTCCGCGCTCA |
| AcsA | CTATGTGAGAGGGCGCGATT | CCTGCACTGCTGTGTTGAAC |
| GltA | TCTAAAAGCTGAGCGCACCA | ATCGCTGCGAAAGTGGAAGA |
| FtnA | AACTTATGCTCAGGCGCACT | TAGATGGCTCGGGGCTTTTC |
| TonB | AACGCTCCCATCAGCATTGA | CTCTGAGGGTGCGACTTCTG |
| FecA | CGCATTCACCGGCTCTCTAT | TGTAGGCACAAGCACGGATT |
